# Perturbation-Modulated Native Mass Spectrometry Excludes a Nonspecific Drug Target Protein Binder Based on Conformation Stability Change

**DOI:** 10.1021/acs.analchem.5c00051

**Published:** 2025-03-18

**Authors:** Xiaobo Tian, Patrick Mueller, Piotr Sosnowski, Fang Li, Dongliang Guan, Charlotte Jacquet, Gérard Hopfgartner

**Affiliations:** † Life Sciences Mass Spectrometry, Department of Inorganic and Analytical Chemistry, University of Geneva, 24 Quai Ernest Ansermet, CH-1211 Geneva 4, Switzerland; ‡ Shandong Laboratory of Yantai Drug Discovery, Bohai Rim Advanced Research Institute for Drug Discovery, Yantai, Shandong 264117, China

## Abstract

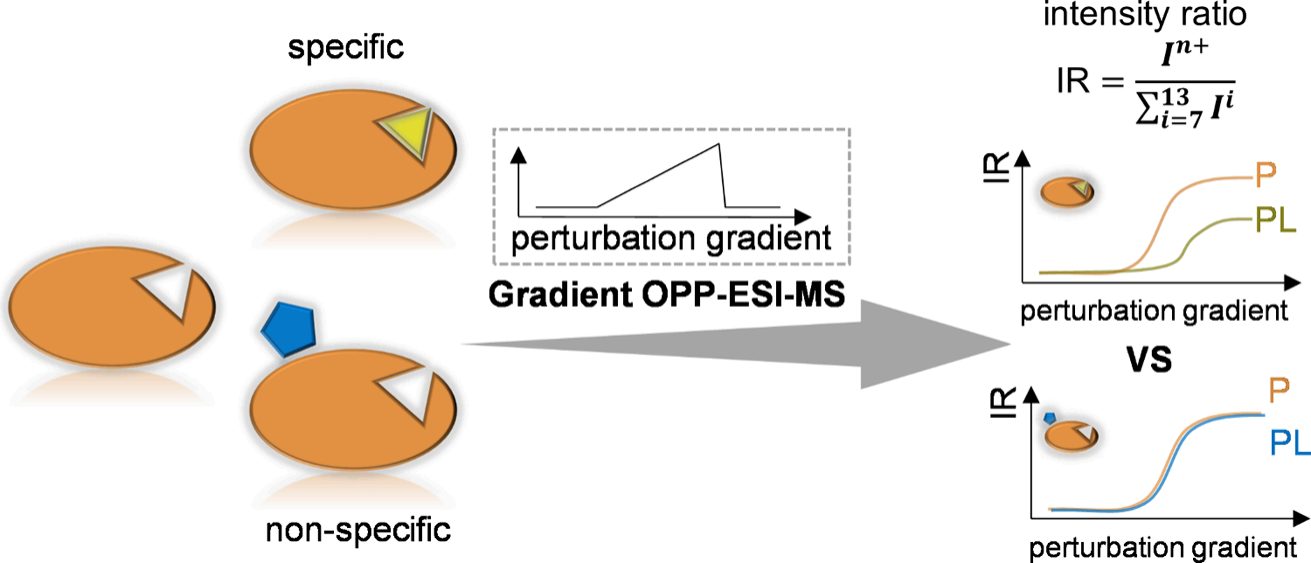

Native electrospray ionization mass spectrometry (ESI-MS) is a standard technique for drug–protein screening but may be affected by nonspecific binding. To address this, a reference protein and ligand must be selected for each protein investigated. We report a versatile, informative, high-throughput screening approach to differentiate specific and nonspecific interactions based on charge state distribution (CSD) changes caused by ionization perturbations (e.g., methanol or heat) without the need for a surrogate. We show that specific binding stabilizes the protein–ligand complex against perturbations, resulting in narrower CSDs compared with the unbound protein. In contrast, no significant difference in CSDs is observed between nonspecific complexes and the free protein. To introduce ion source perturbations without affecting protein–ligand incubation, we employed a 3D-printed open port probe (OPP) that is widely compatible with different instruments and sample introduction techniques. The approach was validated with well-characterized protein–ligand pairs, confirming that cytidine phosphates, triacetylchitotriose, and fluvastatin are specific ligands for ribonuclease A, lysozyme, and beta-lactoglobulin, respectively. Further, cytidine-5′-triphosphate (CTP) was found to interact nonspecifically with lysozyme and beta-lactoglobulin. The approach was applied to screening assays of two drug target proteins, thrombin and dihydrofolate reductase, revealing that for thrombin, fluvastatin may share the same binding site as argatroban, which is supported by competition experiments and molecular docking results. These results provide new insights into the anticoagulation effect of statins and show the potential of the approach in prioritizing candidates for target proteins.

## Introduction

Metabolites, or drugs, produce effects through interactions with biomacromolecules, usually proteins. Thus, investigation of protein–metabolite interactions (PMIs) is essential for deep understanding of the biological mechanism and shows promise in drug discovery for screening hits for the target protein.^1,2^ A variety of MS-based techniques have been reported to study PMIs,^3^ and one widely used method is native electrospray ionization mass spectrometry (ESI-MS),^4^ in which a metabolite or ligand is mixed with the target protein in a native-like solution, usually ammonium acetate (NH_4_Ac), and the mixture is directly introduced to ESI-MS without separation/purification while maintaining the PMIs during ionization. Quantifying the interacting and unbound protein fractions also reveals the stoichiometry and dissociation constants.^8−10^ With the simplicity of sample preparation, low sample consumption, high sensitivity, and high throughput, native ESI-MS is common in drug screening.^1,3^ To optimize native ESI-MS to explore PMIs, a variety of studies have been reported, including the type of ion source,^5,6^ the size of ESI tips,^7^ the buffer maintaining the native conditions,^8,9^ and solvent additives.^6,10−12^ True interactions between proteins and metabolites typically stabilize or lock the protein structure and are the basis for metabolite-centric approaches that identify bound proteins by comparing protein stability with and without the target compound.^13,14^ This difference is even more pronounced in the presence of perturbations, such as heating^15^ and chemical denaturants.^16^

A widely accepted ionization mechanism in native ESI-MS is the charged residue model^9,17^ (CRM), which proposes that charged ESI droplets shrink with solvent evaporation until all solvent molecules are gone. In this process, the concentration of ligands and protein continues to increase, which increases the risk of nonspecific binding compared to physiological conditions.^8,10,11^ Nonspecific binding is structure-independent and usually occurs on the solvent-exposed protein surface, which is consistent with the observed increase with the ligand concentration.^6,7^ Thus, it is likely to be more problematic when measuring weak PMIs, especially since potential hit compounds in early drug screening typically exhibit only medium to low affinities. An excess of ligands is usually required to generate detectable amounts of PMIs, which inadvertently promotes the occurrence of nonspecific binding within ESI. As highlighted in recent reviews on the application of native MS in drug discovery,^2,4^ addressing nonspecific protein ligand binding still largely depends on the reference protein method^10,11^ proposed by Klassen and co-workers in 2006. This approach involves the use of an additional protein (P-ref) that ideally does not interact with the tested protein and ligands as an internal reference, and the fractions of the ligand-bound reference protein are used to correct the true PMIs. However, in practice, it is challenging to select a P-ref that does not have specific interactions with the protein or ligand of interest, particularly in discovery research. Confirming this prerequisite requires validating that the proposed protein indeed exhibits no specific interactions with either the studied protein or the ligand before applying it as a P-ref, which causes additional issues and complexity. The approach also introduces bias due to nonspecific binding between different proteins and ligands, which may vary from case to case. However, no alternative approaches have since been proposed. Only a few studies have dealt with the issue of nonspecific binding between the target protein and the small molecules tested.^6,10,11^ Ye and co-workers^6^ reported differentiating true PMIs from nonspecific binding by acetonitrile-induced changes of the dissociation constant (*K*_d_) and found that *K*_d_ decreased with more acetonitrile in ESI for true PMIs and vice versa for nonspecific binding.

The open port probe (OPP) was first proposed by Van Berkel and Kertesz,^18^ and its application has been explored in various areas.^19^ The core structure of the OPP is based on two coaxial tubes: the outer one receives solvents from a pump, and the inner one aspirates the liquid into the electrospray ion source due to the nebulizing gas forming a liquid cone where the sample can be introduced.^18,20^ Samples can be independently introduced by a pipet, a syringe pump, or an autosampler onto the OPP and are instantly aspirated into the ion source. To simplify OPP integration with different instruments and reduce costs (∼3 euros per piece of OPP), we produced devices with 3D printers and used them for qualitative and quantitative studies.^19,20^

In this study, we report a method that can distinguish true PMIs from the nonspecific binding that is usually observed in native ESI-MS analysis. The method exploits the advantage of the OPP that the solvent can be easily and continuously changed from native (e.g., NH_4_Ac solution) to denaturing (e.g., 50% MeOH solution) conditions without disturbing sample introduction. With this approach, we observed the important phenomenon that stabilization induced by PMIs causes the protein–metabolite complex to show reduced intensity of high charge state ions compared to the unbound protein, which allowed us to identify true PMIs based on alterations in the charge state distribution (CSD). To show the proof-of-concept, we investigated well-studied protein–ligand pairs, such as ribonuclease A–cytidine phosphates, and further applied the approach to assays of the real drug target proteins, thrombin and dihydrofolate reductase (DHFR). This study revealed that in contrast to true PMIs, nonspecific binding between target protein and ligands does not change the CSD compared to the free protein. This approach may serve as a quick second-round check to exclude nonspecific binding after the first-round native-MS assays, thus refining potential candidates to the target protein.

## Experimental Section

The details of chemicals and materials and the parameters for OPP-ESI-MS are provided in the Supporting Information.

### Studies of Protein–Metabolite Interaction under the Native Condition or in the Presence of Perturbations

For infusion experiments, proteins (RNase A, lysozyme, or beta-lactoglobulin) were dissolved in 25 mM NH_4_Ac to a concentration of 100 μM and the corresponding ligands (cytidine phosphates, NTAC, or fluvastatin) were added to a concentration of 1 mM. The mixtures were shaken at room temperature for 5 min before analysis and infused onto the OPP at a rate of 3 μL per min with a syringe pump. For the native condition, the OPP received the same solution of 10 mM NH_4_Ac from an LC pump. For the non-native condition, the OPP received the solutions via an LC gradient: 0–5 min, constant at 10 mM NH_4_Ac; 5–10 min, change from 100% 10 mM NH_4_Ac to 100% H_2_O containing 0.1% FA; 10–20 min, change from 0 to 90% MeOH; 20–25 min, equilibrate at 100% 10 mM NH_4_Ac. For the triplicate analyses, fresh protein–ligand samples were prepared just before the infusion experiment, with a 10 min time interval between each replicate. For stepped OPP-ESI experiments, thrombin and DHFR samples were buffer-exchanged with a cutoff filter (3 kDa) to a concentration of 5 μM in 100 mM NH_4_Ac, followed by screening a set of ligands including Met, Ami, Tri, Pyr, Dab, Arg, Biv, CTP, Flu, and Nor. The ligands were added to 100 μM, and the incubated protein–ligand mixtures were introduced manually by a pipet onto the OPP under 10 mM NH_4_AC, 0.1% FA, or different source temperature conditions (see Table S1 for details).

### Data Analysis

The intensity ratio (IR) for a single charge state in a specific spectrum was calculated by dividing its intensity by the summed intensity of all charge states, IR = *I*^*n*+^/*I*^total^. The collection of ratios for all charges indicates the CSD. The intensity ratios of all charges were plotted versus the gradient, or the CSDs of all charges were displayed in bar graphs against stepped OPP solutions. The binding ratio (BR) was determined by dividing the total intensity (all charge states) of the protein–metabolite by the total intensity of the protein–metabolite [PL] and unbound protein [P], BR = [PL]/([PL] + [P]). For the *K*_d_ evaluation of CTP and CDP to RNase A, the ratio, *R*, of the ligand-bound protein to the free protein was calculated based on intact masses in the deconvoluted spectra that were reconstructed by the embedded Bio Tool Kit in PeakView Software (version 2.2).

## Results and Discussion

### Design and Principle of the Gradient OPP-ESI-MS Approach

**Figure 1:**
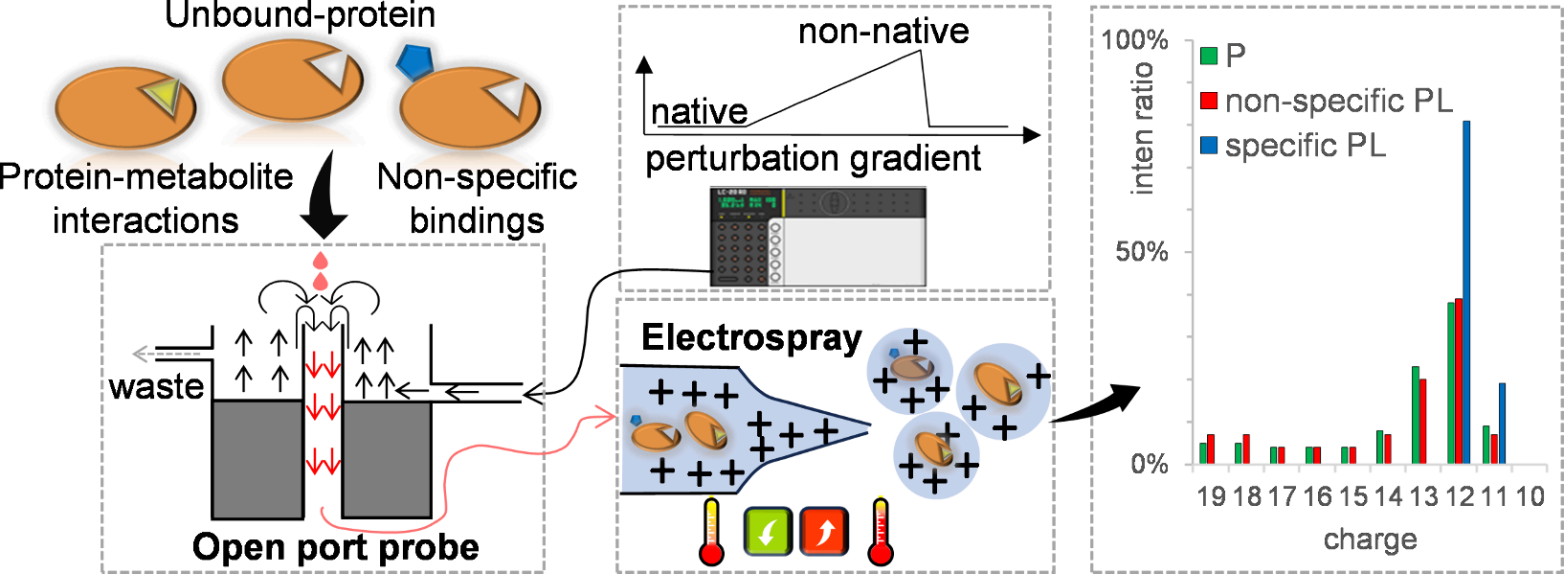
Schematic showing the gOPP-ESI-MS approach for identifying metabolites interacting with a target protein. The system includes an LC pump, the OPP, and an ESI-QqTOF MS. Protein samples with and without ligands are introduced by a pipet or a syringe pump onto the OPP where solutions with native conditions or perturbations, such as MeOH or FA, are delivered by the LC pump. Perturbation with heat is controlled by the ion source temperature. Under different denaturing strengths, the protein and protein–metabolite complex show CSD differences that reveal specific binding.

CSD is widely used to study protein structure changes since an unfolded protein usually has more high charge state ions than its folded forms.^21^ Thus, we reasoned that incorporating denaturing perturbations into native ESI-MS could aid in the identification of metabolites truly interacting with the target protein.^22^ Specifically, we hypothesized that if the perturbations, such as the concentration of MeOH or formic acid (FA) in ESI, were increased, the protein–metabolite complex would show a significant CSD difference compared to the free protein. In contrast, nonspecific interactions are usually on the surface of proteins and should have less influence on structure and hence less effect on the CSD. Here, we describe the gradient OPP-ESI-MS (gOPP-ESI-MS) approach, which differs from conventional native ESI-MS because the OPP allows the ESI solvent to be changed without affecting the protein/protein–metabolite complex incubations. As shown in Figure 1, the protein with and without the ligand is introduced dropwise onto the OPP and instantly aspirated into the ESI source. Increasing the proportion of MeOH/FA in the OPP solvent gradually increases the denaturing effect of the solvent. As a result, proteins will unfold stepwise, and the CSD of the protein will broaden and shift to higher charge states. In addition, due to the stabilization brought by the PMIs, the protein–metabolite complex is expected to be more resistant and show lower intensities in high charge states compared to the unbound protein. Although increasing MeOH/FA could break some PMIs, the intensities of the protein–metabolite ions do not necessarily decrease since the ESI response increases in the presence of MeOH or FA.^23^ Notably, our approach to screening PMIs based on CSDs is not affected by the peak intensities.

### Protein–Metabolite Interaction under Constant Native Conditions with the Open Port Probe

**Figure 2:**
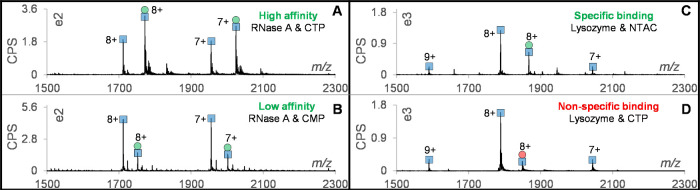
Probing PMIs under the constant native condition. The proteins and ligands are freshly mixed and incubated for 5 min and then dispensed dropwise onto OPP. Incubation of RNase A (100 μM): (A) with CTP (1 mM) and (B) with CMP (1 mM) and incubation of lysozyme (100 μM): (C) with NTAC (1 mM) and (D) with CTP (1 mM). The blue square, green circle, and red circle indicate the unbound protein, the true-positive ligand, and the false-positive ligand, respectively.

We first tested the performance of the gOPP-ESI-MS approach to measure protein metabolite complexes under the native conditions of 10 mM NH_4_Ac. We selected the well-characterized proteins^5,6^ ribonuclease A (RNase A), lysozyme, and beta-lactoglobulin. For RNase A, three known binding ligands, CTP, CDP, and CMP (cytidine triphosphate, diphosphate, and monophosphate), and a nonbinding ligand (cytidine) were separately incubated with RNase A and dispensed dropwise onto the OPP by a syringe pump. As shown in Figure 2A,B, both the unbound RNase A and the three complexes of RNase A with cytidine phosphates exhibited narrow CSDs centered on charges of 7+ and 8+, indicating that they are in the folded state. To simplify the *R* calculation, the ratio of the ligand-bound protein to the unbound protein, charge state deconvolution was performed and *R* was calculated based on intact masses (Figure S1). For CTP, CDP, and CMP, the *R* values are 1.48, 0.60, and 0.31, respectively, which is consistent with the affinity orders reported previously.^5^ No complex was observed for cytidine. The affinity orders were further investigated in competitive and noncompetitive experiments (Figure S2), and the measured orders are consistent with each other. Furthermore, the *K*_d_ of CTP and CDP were measured in titration assays according to the method reported by Daniel et al.,^24^ where the concentration of RNase A was constant and the ligand was measured at a series of concentrations from low to high (Figure S3). The measured *K*_d_ for CTP and CDP were 10.89 and 38.61 μM, respectively, which are slightly higher than the value reported in an earlier study.^5^

To illustrate the issues of nonspecific binding, we selected a true-positive ligand (*N*,*N*′,*N*″-triacetylchitotriose, NTAC) and a false-positive ligand (CTP) for lysozyme.^6^ In Figure 2C,D, the free lysozyme and the complexes with NTAC and CTP are centered on a charge of 8+. The *R* value of NTAC is 0.59, which denotes a relatively strong binding to lysozyme, while the false-positive ligand, CTP, shows low but reliable peaks at the *m*/*z* of lysozyme-CTP, with a *R* value around 0.05 (Figure 2D). Further, we investigated the nonspecific binding of CTP and beta-lactoglobulin and compared it with fluvastatin, which was reported to specifically bind to beta-lactoglobulin^25^ (Figure S1G,H). From these data, it is clear that based solely on the mass shifts, we can not exclude CTP as a real ligand for lysozyme and beta-lactoglobulin, and additional methods are needed.

### Charge State Distribution Changes with Open Port Probe Solvent Composition

**Figure 3:**
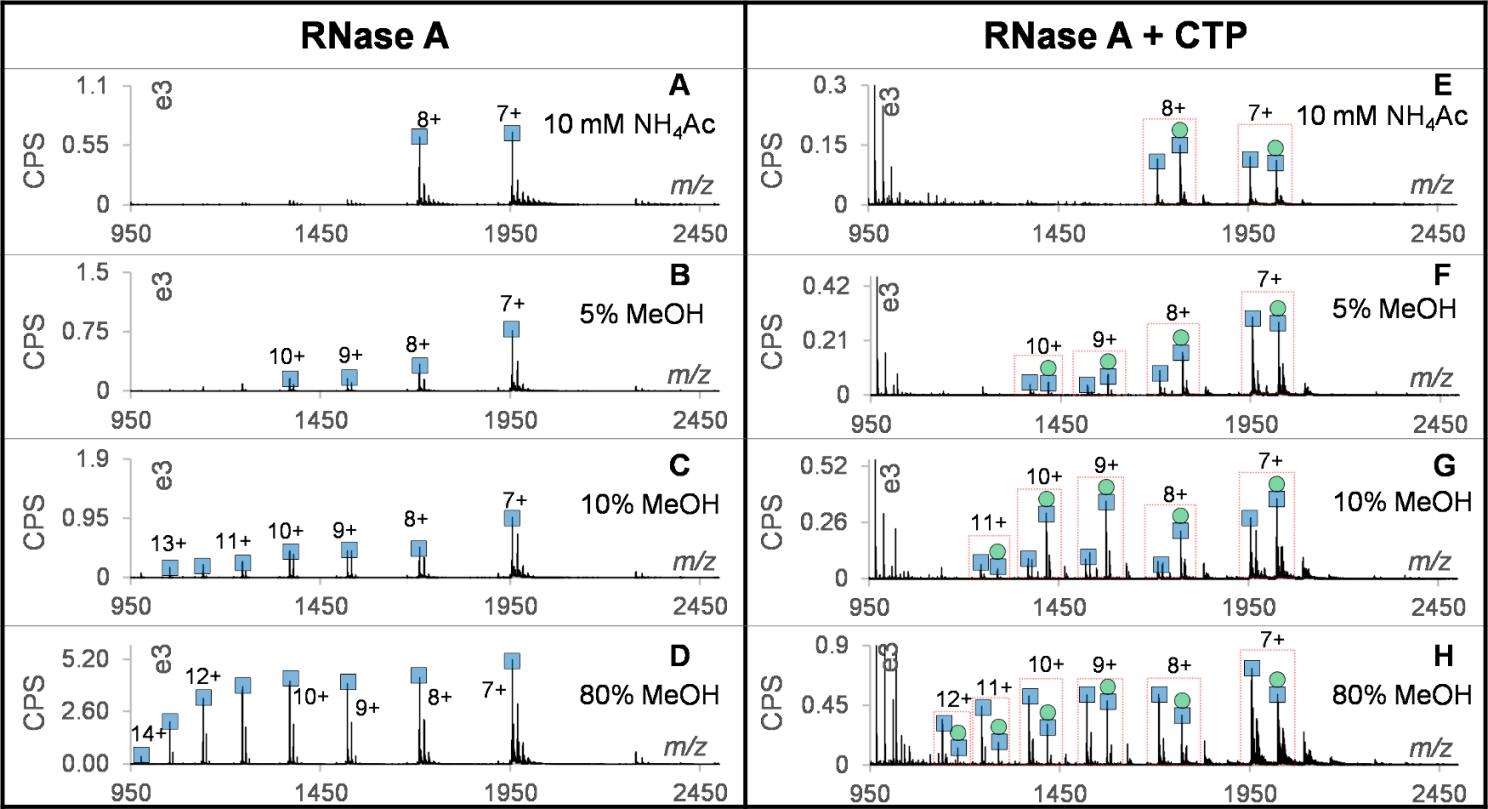
Changing CSD of RNase A due to the gradient of the OPP solvent. Spectra of RNase A at various proportions of MeOH: (A) 100% 10 mM NH_4_Ac, (B) 5% MeOH, (C) 10% MeOH, and (D) 80% MeOH. Spectra of CTP-bound RNase A and/or unbound RNase A at various proportions of MeOH: (E) 100% 10 mM NH_4_Ac, (F) 5% MeOH, (G) 10% MeOH, and (H) 80% MeOH. The “double peak” in panels (C) and (D) are adducts with phosphate (*m*/*z* shift of 98.3). The blue squares and green circles represent the unbound protein and CTP binding, respectively.

The core finding in this work is that we can differentiate true PMIs from nonspecific binding by comparing changes in the CSD of the free protein and protein–metabolite complex versus the increasing amount of MeOH/FA. To modulate CSD from native to unfolded state, solutions of RNase A and myoglobin were continuously infused onto the OPP while the OPP solvent was changed from 100% NH_4_Ac to 100% H_2_O containing 0.1% FA, followed by a MeOH gradient (final value 90%) with an LC pump. Overall, the intensities of protein peaks increased more than 5-fold over the gradient as shown by the base peak chromatographs (BPCs) in Figures S4 and S5A. The CSD of native RNase A was centered on charges of 7+ and 8+ under 100% 10 mM NH_4_Ac (Figure 3A), while charges of 9+ and 10+ appeared around 5% MeOH (Figure 3B), followed by the higher charge states (10+ to 14+), which became more intense with increasing MeOH (Figure 3C,D). To show the CSD changes induced by ligand binding, the complex of RNase A with CTP was investigated under the same conditions (Figure 3E–H). In 10 mM NH_4_Ac, the charge states remained centered on charges of 7+ and 8+ (Figure 3E), while with the higher proportion of MeOH, the complex of RNase A with CTP showed changes in intensity ratios for charges 10+, 11+, and 12+ (Figure 3H). An interesting phenomenon was the formation of phosphate adducts on RNase A with higher MeOH, which was also reported in a previous work.^26^ Further, we investigated another model protein, myoglobin, that naturally contains the ligand heme.^6^ In 10 mM NH_4_Ac, the CSD of the heme-bound myoglobin centered on charges 8+ and 9+ and almost no free heme was observed (Figure S5B). With the solvent changed to 100% H_2_O containing 0.1% FA, heme-bound myoglobin and unbound myoglobin were observed, and we estimated that more than 50% of the complexes are already dissociated by the presence of FA (Figure S5C). However, the free heme did not obviously increase, and we speculate that this is because the 100% aqueous solution of the OPP is not appropriate for the ionization of heme (Figure S5 B vs C). In contrast, with higher levels of MeOH (Figure S5D,E), the intensity of heme increased significantly (Figure S5 C vs E). The appearance of a broad CSD and high charge states reveals the unfolding of the protein that makes more ionization sites accessible and results in a gradual increase in spectrum intensity.

### Repeatability Evaluation and CSD Differences between the Unbound and CDP-Bound RNase A

To evaluate the repeatability of the gOPP-ESI-MS approach, triplicate analyses of the interaction between CDP and RNase A were investigated under two conditions: constant 10 mM NH_4_Ac (native conditions) and a gradient ranging from native to non-native conditions. Around 38% of RNase A was bound with CDP and the ratios remained unchanged in 10 mM NH_4_Ac for more than 25 min, which implies that the sample introduction by syringe and OPP-ESI ionization are reproducible enough for further analysis (Figure S6). In addition, the intensity ratios of all charge states remained unchanged under native conditions (Figure S7). For analyses under the gradient (Figure S8), the binding ratio started to decrease around 8 min (at 40% 10 mM NH_4_Ac and 60% H_2_O containing 0.1% FA) and stayed unchanged even with 90% MeOH. The three replicates overlapped each other, indicating that changing the OPP solution does not influence reproducibility.

**Figure 4:**
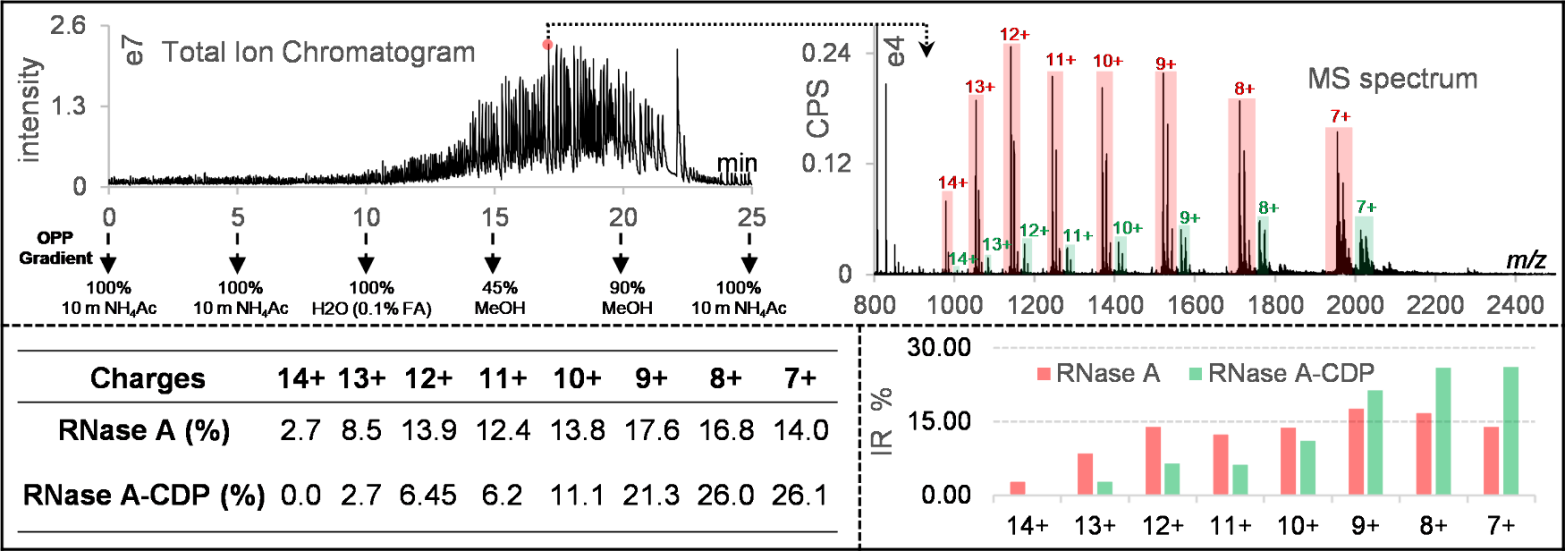
CSD calculation example. The MS spectrum was taken at a point around 70% MeOH. The RNase A–CDP complex and unbound-RNase A showed broad but different CSDs.

Unlike the binding ratio, the intensity ratios of most charge states did not change significantly before 10 min. This suggests the need for stronger perturbations to reveal the CSD difference and is supported by the appearance of charge states 11+ to 14+ after 10 min with maxima of ca. 17 min (Figure S9). The antidenaturing effect of PMIs is exemplified by the spectrum taken at 70% MeOH, which shows the induced CSD difference between RNase A & CDP complex and unbound-RNase A (Figure 4). To distinguish the CSD differences caused by PMIs, we named the RNase A in the control sample without CDP as “control-RNase A” and the unbound RNase A in the experimental sample containing both RNase A and CDP as “unbound-RNase A”. The CSD of control-RNase A and unbound-RNase A almost exactly overlap during the gradient, indicating that the excess free ligand (CDP) in the experimental sample did not affect the CSD of RNase A (Figure S9). Consequently, we can confidently infer that changes in the CSD of the RNase A & CDP complex are due to their interactions, which may tighten/lock the structure of RNase A and alter the accessibility of ionization sites. As we expected, the RNase A & CDP complex did show significant differences in CSD compared to the unbound-RNase A. In particular, the distinction is most pronounced for charge states 12+ and 13+, which appear approximately 3 min later in the complex and have lower intensity ratios than for unbound-RNase A.

### Differentiating True PMIs and Nonspecific Binding

**Figure 5:**
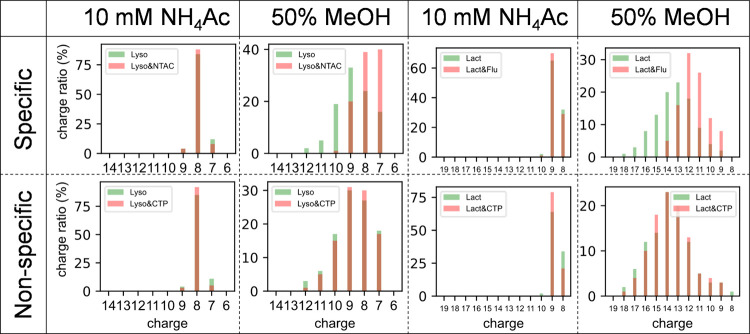
Differentiations between the true PMIs and nonspecific bindings. The comparison of CSDs for lysozyme–NTAC with lysozyme–CTP and beta-lactoglobulin–fluvastatin with beta-lactoglobulin–CTP.

To demonstrate the effectiveness of the gOPP-ESI-MS approach in differentiating between true PMIs and nonspecific binding, we studied lysozyme and beta-lactoglobulin with known true-positive and false-positive ligands. Consistent with a previous report,^6^ the true PMIs of lysozyme–NTAC and beta-lactoglobulin–fluvastatin showed a downward trend for binding ratios with higher concentration of MeOH (Figures S10 and S11). In contrast, the nonspecific binding of lysozyme–CTP and beta-lactoglobulin–CTP were unchanged or even slightly increased. In addition, we noted that the true PMIs show distinct changes in CSD compared to the unbound protein, e.g., the comparison of raw spectra taken at 10 mM NH_4_Ac and 50% MeOH of OPP solutions (Figure S12). Additional CSD comparisons at six OPP solutions are shown for lysozyme in Figure S13 and for beta-lactoglobulin in Figure S14. Under native conditions (10 mM NH_4_Ac), Figure 5, the CSDs of unbound protein and protein and ligand complex are similar, which is attributed to the folded status of the protein. This indicates that perturbation is needed to reveal the differences; since the denaturing strength increases with increasing FA and/or MeOH concentration, the protein should gradually unfold and produce more intense high charge state ions. In Figure 5, both the unbound proteins and the nonspecific binding with CTP showed broader CSDs under 50% MeOH, while the specific binding between lysozyme & NTAC and beta-lactoglobulin and fluvastatin maintained narrower CSDs owing to tightening of the protein structure. Plots for specific charge states (Figures S15 and S16) show that for low charge states, e.g., 9+ for lysozyme and 10+ for beta-lactoglobulin, true PMIs and nonspecific binding have similar patterns, while the intensity ratio differences are greatly pronounced for high charge states, such as 11+ and 12+ for lysozyme and 12+ and 17+ for beta-lactoglobulin. Compared to nonspecific binding, high charge states of true PMIs require high proportions of MeOH and are less intense. An interesting phenomenon was observed for beta-lactoglobulin where charges 11+ to 13+ appeared at 10 min (100% H_2_O containing 0.1% FA) while 14+ to 18+ appeared at 15 min (45% MeOH) (Figure S16). The distinct changes suggest that there are two unfolding stages and might be useful for structural biology research.

### Screening of Thrombin Interactions

**Figure 6:**
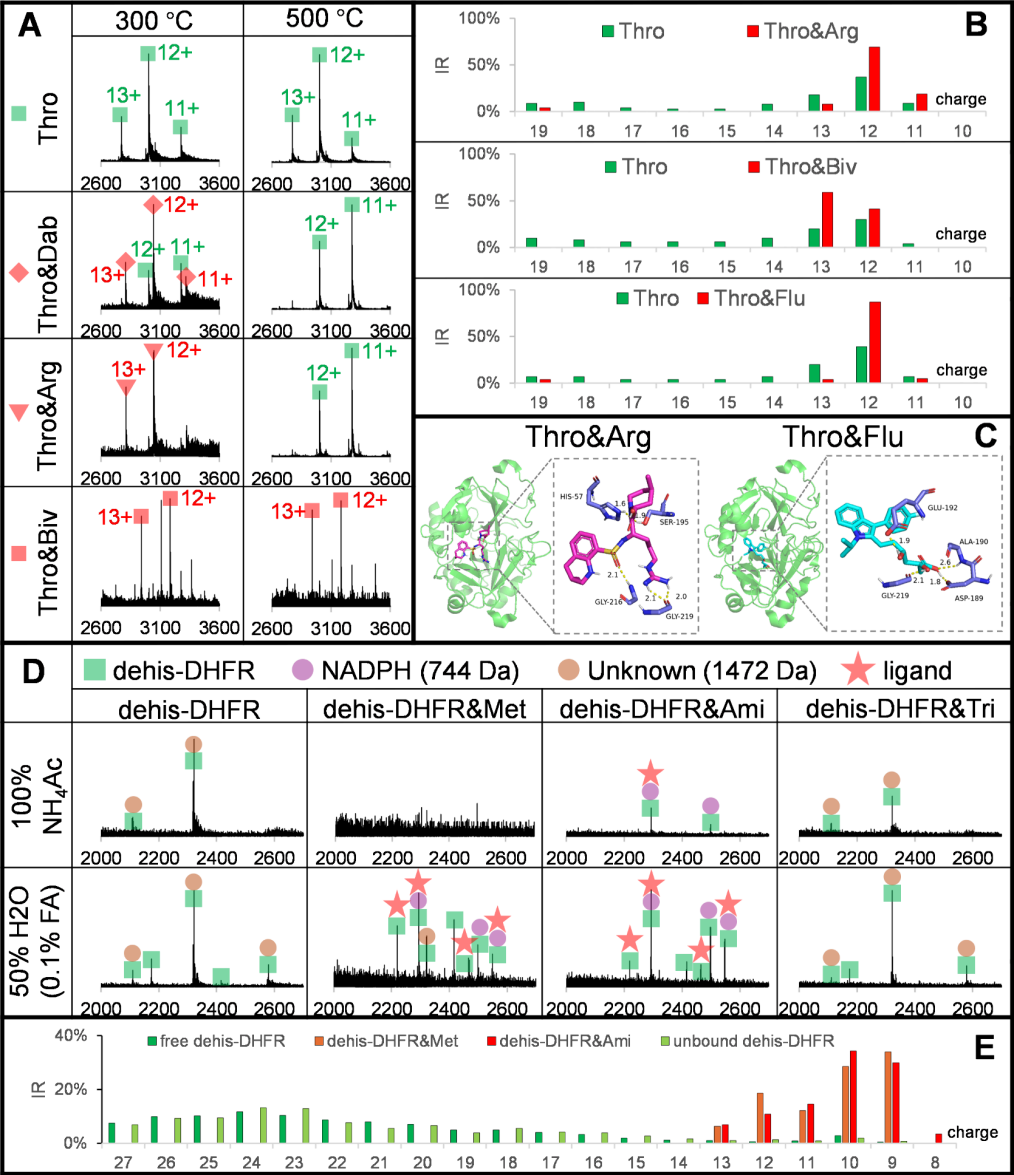
Screening thrombin and DHFR with gOPP-ESI-MS under conditions 1 to 6 (see conditions in Table S1). (A) Raw mass spectra of free thrombin, thrombin and dabigatran, thrombin and argatroban, and thrombin and bivalirudin of screenings under condition 1 (left) and condition 2 (right); (B) CSDs of thrombin with argatroban, bivalirudin, and fluvastatin under condition 3; (C) docking model of thrombin binding with argatroban and fluvastatin. Key residues involved in the interactions with the ligand are shown as stick models. (D) Raw mass spectra of free dehis-DHFR, dehis-DHFR-methotrexate, dehis-DHFR-aminopterin, and dehis-DHFR-trimethoprim from screening under conditions 4 and 6; (E) the CSDs of free dehis-DHFR, dehis-DHFR-methotrexate, dehis-DHFR-aminopterin, and unbound dehis-DHFR under OPP condition 5.

Thrombin, a serine protease, influences the blood coagulation cascade in various ways and is a target for anticoagulant medications, such as bivalent (bivalirudin) and direct small molecule inhibitors (dabigatran and argatroban). Studying thrombin–ligand interactions may provide insights into developing new inhibitors with fewer bleeding-related adverse effects. Thrombin was screened against 10 compounds (Figure S17) under 3 different OPP conditions (Table S1, conditions 1–3). Under the 10 mM NH_4_AC and 300 °C condition, (Figure S18A), three thrombin inhibitors dabigatran, argatroban, and bivalirudin showed good binding ratios. Free thrombin showed a narrow CSD centered on charges 11+, 12+, and 13+ (Figure 6A), respectively, while argatroban and bivalirudin showed 100% binding with thrombin and generated CSDs mainly centered on charges 12+ and 13+, respectively. Thrombin & dabigatran showed 91% binding, but with additional peaks corresponding to free thrombin with a different CSD, i.e., the absence of charge 13+ and a different ratio for charges 11+ and 12+, which were hypothesized to arise from dissociation induced by heating in the ion source. This was confirmed by screening at 500 °C (Figure 6A right panel), where dabigatran and argatroban showed peaks for 11+ and 12+ free thrombin without protein–ligand peaks, while bivalirudin lacked these dissociation peaks, probably due to the bivalent inhibitor binding at the active site and exosite I simultaneously. Accordingly, we rank the heat susceptibility as dabigatran > argatroban > bivalirudin. Even through dissociation was unexpected at 500 °C, binding with dabigatran and argatroban had already induced significant CSD changes from the free thrombin. Thrombin was further screened under the 10 mM NH_4_AC, 0.1% FA (50/50%), 300 °C condition, where complexes of argatroban and bivalirudin partially dissociated but the remaining complexes showed different CSD to free thrombin (CSD in Figure 6B, raw spectra in Figure S19). These observations authenticated argatroban and bivalirudin as specific inhibitors to thrombin. Similarly, with the 10 mM NH_4_AC and 500 °C condition, more than 98% of the dabigatran complex dissociated under 50% H_2_O containing 0.1% FA, but the FA-dissociated thrombin showed the same CSD as the free protein (Figure S20). This may suggest that the dissociation induced by heat and acid is different and highlights that perturbations applied to different proteins should be carefully tailored. Further, we observed that the complexes of argatroban and bivalirudin showed different CSD, which could result from different binding mechanisms and may allow allosteric binders for a specific target protein to be explored.

However, the more interesting finding is that fluvastatin showed around 10% binding under the 10 mM NH_4_AC and 300 °C OPP condition (Figure S18A), dissociation under the 10 mM NH_4_AC and 500 °C OPP condition (Figure S17), and a distinct CSD to the free thrombin under the 10 mM NH_4_AC, 0.1% FA (50/50%), 300 °C OPP condition (Figure 6B), which suggests that fluvastatin may also bind specifically with thrombin. To investigate further, competition binding assays were performed where dabigatran and argatroban were added separately and together to the incubation of fluvastatin and thrombin under the 10 mM NH_4_AC and 300 °C condition. Fluvastatin showed around 10% binding with thrombin, which completely disappeared after adding dabigatran, argatroban, or both (Figure S18B). Further, in all competitive experiments, only the complexes of “one ligand to one protein” were found, which confirmed that fluvastatin, dabigatran, and argatroban bind with thrombin at the same active site and that fluvastatin is replaced with dabigatran or argatroban from the initial fluvastatin and thrombin complex. To obtain a direct three-dimensional view of the binding between thrombin and fluvastatin, we conducted molecular docking of argatroban (positive control) and fluvastatin with thrombin (PDB ID: 3VXF)^27^ (Figure 6C). The results indicated that argatroban could bind to the active site of thrombin, forming hydrogen bonds with surrounding residues His57, Ser195, Gly216, and Gly219 and hydrophobic interactions with Trp60D, Leu99, Ile174, and Trp215, thus maintaining good binding. Similarly, fluvastatin could also bind to the active site of thrombin, forming hydrogen bonds with Asp189, Ala190, Glu192, and Gly219 and hydrophobic interactions with Trp60D, Leu99, and Trp215 (Figure S21). These findings support our hypothesis that fluvastatin binds with thrombin at the same active site as argatroban but has less affinity and may provide insights into understanding how statins work in anticoagulation.^28^

### Screening of Dihydrofolate Reductase (DHFR) Interactions

Human DHFR is a target for cancer treatment as inhibition of DHFR can limit production of tetrahydrofolate that is essential for the growth and proliferation of tumor cells. Recombinant human DHFR was screened against 10 ligands under two OPP solvent conditions (Table S1, conditions 4–5) selected based on rapid assessment of OPP solutions with a gradient (Figure S22). Only the human DHFR inhibitors methotrexate and aminopterin showed 100% DHFR binding (Figure S23); no other ligands, not even the bacterial DHFR inhibitors trimethoprim and pyrimethamine, bound to human DHFR. In the measurements of DHFR with argatroban and bivalirudin, no protein signal was observed due to ionization suppression (Figure S24). Although we observed free DHFR and the complexes with methotrexate and aminopterin (Figure S25), all peaks appeared as pairs, which reduced the *S*/*N* ratio and complicated data analysis. The splitting was determined to arise from a gluconoyl modification (178 Da) on the his-tag, which commonly occurs on his-tagged recombinant proteins expressed in *Escherichia coli*.^29^ Unexpectedly, in addition to the added ligand, we observed two small molecules that form a binary or ternary complex with DHFR in the presence of the corresponding ligand, which further complicated spectra. These small molecules were not removed during the buffer exchange with a cutoff filter, indicating that they had interacted with DHFR. One was determined to be the cofactor NADPH^30^ (744 Da), and the other is unknown and has a mass of 1472 Da. Interestingly, we found that the amino acid sequence of recombinant human DHFR includes a thrombin cleavage site (-LVPRGS-) before the his-tag site (Figure S26). To simplify spectra, the his-tag on the recombinant human DHFR was enzymatically cleaved with thrombin, and the loss was efficiently and specifically finished in 5 h, generating dehis-DHFR (Figure S27). As shown in the top part of Figure 6D, the peak splitting disappeared, leaving two clear charge peaks in the spectrum of free dehis-DHFR. However, the peak intensity did not double as expected, which could be explained by a reduction of DHFR ionization sensitivity due to loss of the his-tag. This issue was more serious for the complex of dehis-DHFR with methotrexate and aminopterin since the peak intensity dropped severely or disappeared compared to the same experiments with DHFR (Figure S25). We speculate that methotrexate and aminopterin still have good binding with dehis-DHFR, but structure tightening induced by ligand binding depressed accessible ionization sites. This is shown in the bottom panel of Figure 6D, screenings under 10 mM NH_4_AC, and 0.1% FA (50/50%) at 400 °C, in which both dehis-DHFR complexes with methotrexate and aminopterin showed clear binding as ternary (dehis-DHFR & NADPAH & ligand) or binary (dehis-DHFR and ligand) complexes. These findings indicate that the his-tag does not affect ligand binding, and the presence of FA in OPP solution significantly enhances ionization, albeit while slightly disrupting binding.

The complexes of dehis-DHFR with ligands were further screened under the OPP solvent condition 100% H_2_O containing 0.1% FA. In Figure 6E, free and unbound dehis-DHFR showed more intensity for high charges (15+ to 27+). In contrast, the complexes with methotrexate and aminopterin produced narrow CSDs centered on charges 9+ to 13+, indicating their specific interactions with dehis-DHFR. In the comparison between DHFR and dehis-DHFR binding with aminopterin (Figure S28), we noticed that the unknown binder with mass 1472 Da also counteracted unfolding to some extent (Figure S28 left panels). Further, we found that methotrexate and aminopterin facilitate the association of DHFR-NADPH and antagonize the unknown binder with a mass of 1472 Da. From the assays of dehis-DHFR, it is notable that 100% NH_4_Ac buffer is not always the ideal solution to discover binding partners for a specific protein. Introducing perturbations, such as FA or MeOH, not only improves mass spectra quality but also makes the ligand-induced alteration more pronounced to discern specific ligands.

## Conclusions

In summary, prompted by the need to distinguish nonspecific protein–ligand binding, we propose introducing perturbations such as organic solvents, chemical denaturants, acids, bases, or temperature into the study of PMIs by native ESI-MS. We verified that true PMIs preserve the structure in the presence of perturbations, manifesting as the delayed occurrence and/or reduced intensity of higher charge states compared to free protein or nonspecific complexes. The approach was implemented with a 3D-printed OPP system coupled with ESI-MS, and we found that true PMIs and nonspecific binding have distinctive CSD behavior under conditions changing from native to non-native allowing us to screen the true PMIs. The differentiation between true-positive and false-positive ligands was exemplified with well-studied protein–ligand pairs, including RNase A, lysozyme, and beta-lactoglobulin and the corresponding ligands. The approach was further demonstrated with screening assays against 10 ligands of drug target proteins, thrombin, and DHFR. Notably, fluvastatin may bind at the same active site of thrombin as argatroban, and this finding is supported by the competition experiments and confirmed by molecular docking results. The gOPP-ESI-MS approach can be used as a quick second-round assay to exclude nonspecific binding after conventional native ESI-MS experiments, and we believe that this approach holds the promise of avoiding false-positive ligands, thus further narrowing the range of drug candidates for the target proteins. Our gOPP-ESI-MS approach allows for the optimization of perturbation conditions in just minutes using micrograms of protein. Moreover, while a syringe pump and pipet were used for sample introduction in this work, 3D-printed OPP can be readily integrated with fully automated sample introduction devices, such as CTC sample injector and Echo MS,^31^ to enable middle- to high-throughput analysis.

## Supporting Information Available

Details on chemicals and materials, the parameters for OPP-ESI-MS, Table S1, and Figures S1–S28 (PDF)

## References

[ref1] Gavriilidou A. F. M.; Sokratous K.; Yen H.-Y.; De Colibus L. High-Throughput Native Mass Spectrometry Screening in Drug Discovery. Front. Mol. Biosci. 2022, 9, 837901 10.3389/fmolb.2022.837901. 35495635 PMC9047894

[ref2] Cui M.; Du Y. TrAC Trends in Analytical Chemistry 2024, 174, 117701 10.1016/j.trac.2024.117701.

[ref3] Woods L. A.; Dolezal O.; Ren B.; Ryan J. H.; Peat T. S.; Poulsen S.-A. J. Med. Chem. 2016, 59, 2192–2204. 10.1021/acs.jmedchem.5b01940. 26882437

[ref4] Bennett J. L.; Nguyen G. T. H.; Donald W. A. Chem. Rev. 2022, 122, 7327–7385. 10.1021/acs.chemrev.1c00293. 34449207

[ref5] Liu P.; Zhang J.; Ferguson C. N.; Chen H.; Loo J. A. Anal. Chem. 2013, 85, 11966–11972. 10.1021/ac402906d. 24237005 PMC3901310

[ref6] Zheng Q.; Tian Y.; Ruan X.; Chen H.; Wu X.; Xu X.; Wang G.; Hao H.; Ye H. Anal. Chim. Acta 2018, 1036, 58–65. 10.1016/j.aca.2018.07.072. 30253837

[ref7] Báez Bolivar E. G.; Bui D. T.; Kitova E. N.; Han L.; Zheng R. B.; Luber E. J.; Sayed S. Y.; Mahal L. K.; Klassen J. S. Anal. Chem. 2021, 93, 4231–4239. 10.1021/acs.analchem.0c05003. 33630563

[ref8] Konermann L.; Liu Z.; Haidar Y.; Willans M. J.; Bainbridge N. A. Anal. Chem. 2023, 95, 13957–13966. 10.1021/acs.analchem.3c02546. 37669319

[ref9] Hedges J. B.; Vahidi S.; Yue X.; Konermann L. Anal. Chem. 2013, 85, 6469–6476. 10.1021/ac401020s. 23724896

[ref10] Sun J.; Kitova E. N.; Wang W.; Klassen J. S. Anal. Chem. 2006, 78, 3010–3018. 10.1021/ac0522005. 16642987

[ref11] Sun N.; Soya N.; Kitova E. N.; Klassen J. S. J. Am. Soc. Mass Spectrom. 2010, 21, 472–481. 10.1016/j.jasms.2009.12.002. 20089416

[ref12] Cubrilovic D.; Zenobi R. Anal. Chem. 2013, 85, 2724–2730. 10.1021/ac303197p. 23347283

[ref13] Savitski M. M.; Reinhard F. B. M.; Franken H.; Werner T.; Savitski M. F.; Eberhard D.; Molina D. M.; Jafari R.; Dovega R. B.; Klaeger S.; Kuster B.; Nordlund P.; Bantscheff M.; Drewes G. Tracking cancer drugs in living cells by thermal profiling of the proteome. Science 2014, 346, 1255784 10.1126/science.1255784. 25278616

[ref14] Piazza I.; Kochanowski K.; Cappelletti V.; Fuhrer T.; Noor E.; Sauer U.; Picotti P. A Map of Protein-Metabolite Interactions Reveals Principles of Chemical Communication. Cell 2018, 172, 358–372. 10.1016/j.cell.2017.12.006. 29307493

[ref15] Franken H.; Mathieson T.; Childs D.; Sweetman G. M. A.; Werner T.; Tögel I.; Doce C.; Gade S.; Bantscheff M.; Drewes G.; Reinhard F. B. M.; Huber W.; Savitski M. M. Nat. Protoc. 2015, 10, 1567–1593. 10.1038/nprot.2015.101. 26379230

[ref16] West G. M.; Tucker C. L.; Xu T.; Park S. K.; Han X.; Yates J. R.; Fitzgerald M. C. Proc. Natl. Acad. Sci. U. S. A. 2010, 107, 9078–9082. 10.1073/pnas.1000148107. 20439767 PMC2889096

[ref17] Kebarle P.; Verkerk U. H. Mass Spectrom. Rev. 2009, 28, 898–917. 10.1002/mas.20247. 19551695

[ref18] Van Berkel G. J.; Kertesz V. Rapid Commun. Mass Spectrom. 2015, 29, 1749–1756. 10.1002/rcm.7274. 26331924

[ref19] Sosnowski P.; Marin V.; Tian X.; Hopfgartner G. Analyst 2022, 147, 4318–4325. 10.1039/D2AN00925K. 36040388

[ref20] Sosnowski P.; Hopfgartner G. Talanta 2020, 215, 120894 10.1016/j.talanta.2020.120894. 32312439

[ref21] Grandori R. J. Mass Spectrom 2003, 38, 11–15. 10.1002/jms.390. 12526001

[ref22] Luo P.; Liu Z.; Lai C.; Jin Z.; Wang M.; Zhao H.; Liu Y.; Zhang W.; Wang X.; Xiao C.; Yang X.; Wang F. J. Am. Chem. Soc. 2024, 146, 8832–8838. 10.1021/jacs.4c00316. 38507251

[ref23] Liigand J.; de Vries R.; Cuyckens F. Rapid Commun. Mass Spectrom. 2019, 33, 314–322. 10.1002/rcm.8352. 30440111

[ref24] Daniel J. M.; Friess S. D.; Rajagopalan S.; Wendt S.; Zenobi R. Int. J. Mass Spectrom. 2002, 216, 1–27. 10.1016/S1387-3806(02)00585-7.

[ref25] Barbiroli A.; Beringhelli T.; Bonomi F.; Donghi D.; Ferranti P.; Galliano M.; Iametti S.; Maggioni D.; Rasmussen P.; Scanu S.; Vilardo M. C. Bovine β-lactoglobulin acts as an acid-resistant drug carrier by exploiting its diverse binding regions. Biol. Chem. 2010, 391, 21–32. 10.1515/bc.2010.008. 19919177

[ref26] Zhang S.; Van Pelt C. K.; Wilson D. B. Anal. Chem. 2003, 75, 3010–3018. 10.1021/ac034089d. 12964745

[ref27] Yamada T.; Kurihara K.; Ohnishi Y.; Tamada T.; Tomoyori K.; Masumi K.; Tanaka I.; Kuroki R.; Niimura N. Biochimica et Biophysica Acta (BBA) - Proteins and Proteomics 2013, 1834, 1532–1538. 10.1016/j.bbapap.2013.05.014. 23712263

[ref28] Sekiya A.; Morishita E.; Maruyama K.; Torishima H.; Ohtake S. Journal of Atherosclerosis and Thrombosis 2015, 22, 660–668. 10.5551/jat.28175. 25735397

[ref29] Geoghegan K. F.; Dixon H. B.; Rosner P. J.; Hoth L. R.; Lanzetti A. J.; Borzilleri K. A.; Marr E. S.; Pezzullo L. H.; Martin L. B.; LeMotte P. K.; McColl A. S.; Kamath A. V.; Stroh J. G. Anal. Biochem. 1999, 267, 169–184. 10.1006/abio.1998.2990. 9918669

[ref30] Cammarata M.; Thyer R.; Lombardo M.; Anderson A.; Wright D.; Ellington A.; Brodbelt J. S. Chemical Science 2017, 8, 4062–4072. 10.1039/C6SC05235E. 29967675 PMC6020862

[ref31] Van Puyvelde B.; Hunter C. L.; Zhgamadze M.; Savant S.; Wang Y. O.; Hoedt E.; Raedschelders K.; Pope M.; Huynh C. A.; Ramanujan V. K.; Tourtellotte W.; Razavi M.; Anderson N. L.; Martens G.; Deforce D.; Fu Q.; Dhaenens M.; Van Eyk J. E. Acoustic ejection mass spectrometry empowers ultra-fast protein biomarker quantification. Nat. Commun. 2024, 15, 5114 10.1038/s41467-024-48563-z. 38879593 PMC11180209

