# Supplementary material for: Perturbation-Modulated Native Mass Spectrometry Excludes a Nonspecific Drug Target Protein Binder Based on Conformation Stability Change
Source: Anal Chem. 2025 Mar 18;97(12):6762–70. doi: 10.1021/acs.analchem.5c00051 (PMC11966596; doi:10.1021/acs.analchem.5c00051)
Supplement: Supplementary file 1 — ac5c00051_si_001.pdf [file ac5c00051_si_001.pdf]

Supporting information

Perturbation-modulated native mass spectrometry excludes non-specific drug target protein binder based on conformation stability change

Xiaobo Tian,[†] Patrick Mueller,[†] Piotr Sosnowski,[†] Fang Li,[‡] Dongliang Guan,[‡] Charlotte Jacquet,[†] Gérard Hopfgartner*[†]

[†] Life Sciences Mass Spectrometry, Department of Inorganic and Analytical Chemistry, University of Geneva, 24 Quai Ernest Ansermet, CH-1211 Geneva 4, Switzerland

[‡] Shandong Laboratory of Yantai Drug Discovery, Bohai Rim Advanced Research Institute for Drug Discovery, Yantai, Shandong 264117, China

Corresponding Author *E-mail: gerard.hopfgartner@unige.ch

Table of Contents

Table S1. Conditions applied in screening assays of thrombin and human DHFR 5

Figure S1. Probing PMIs under the constant native condition of 10 mM NH_4Ac 6

Figure S2. Ranking the affinity of CTP, CDP, and CMP to RNase A. 7

Figure S3. Measuring the Kd of CTP and CDP to RNase A by titration assays................. 8

Figure S4. The BPCs of RNase A over the gradient from 0 to 90% MeOH........................ 9

Figure S5. Modulating CSD of myoglobin by increasing the proportion of methanol in the OPP solvent 10

Figure S6. Reproducibility of the gOPP-ESI-MS approach under native condition. 11

Figure S7. Triplicates analyses of the interaction between CDP and RNase A under the native conditions. 12

Figure S8. Reproducibility of the gOPP-ESI-MS approach under a gradient. 13

Figure S9. Triplicates analyses of the interaction between CDP and RNase A under a gradient 14

Figure S10. Binding ratio differentiations between the true PMIs and non-specific bindings. 15

Figure S11. Binding ratio differentiations between the true PMIs and non-specific bindings 15

Figure S12. Differentiations between the true PMIs and non-specific bindings. 15

Figure S13. CSD comparation (lysozyme).. 16

Figure S14. CSD comparation (beta-lactoglobulin) 17

Figure S15. Comparation between the true PMIs (lysozyme-NTAC) and nonspecific binding (lysozyme-CTP) in CSD over the gradient...................................... 18

Figure S16. Comparation between the true PMIs (beta-lactoglobulin-fluvastatin) and nonspecific binding (beta-lactoglobulin-CTP) in CSD over the gradient 19

Figure S17. Raw mass spectra of thrombin incubated with 10 ligands under three conditions. 25

Figure S18. Screening thrombin and competition experiments under conditions 1:....... 26

Figure S19. Raw mass spectra of thrombin & argatroban, thrombin & bivalirudin, and thrombin & fluvastatin.. 27

Figure S20. Raw mass spectra and CSD of free thrombin and the complex of thrombin & dabigatran under condition 3 27

Figure S21. Original outputs of dockings of argatroban versus fluvastatin 28

Figure S22. Rapid assessment of OPP solutions with a gradient. 29

Figure S23. DHFR binding ratios of screenings 30

Figure S24 Original mass spectra of screening DHFR against 10 ligands under condition 1 40

Figure S25. Raw mass spectra of free DHFR, DHFR & methotrexate, DHFR & aminopterin, and DHFR & trimethoprim of screenings under condition 1: 100% 10 mM NH4Ac. 41

Figure S26. Amino acid sequence of the recombinant human DHFR. 42

Figure S27. Tracking the loss of the his-tag from recombinant human DHFR with thrombin. 43

Figure S28. Raw mass spectra of free DHFR, DHFR & aminopterin, dehis-DHFR and dehis-DHFR & aminopterin under condition 6 44

Experimental Section

Chemicals and Materials. Ribonuclease A (RNase A), lysozyme and beta-lactoglobulin, *N, N′, N″*-triacetylchitotriose (NTAC), cytidine-5'-triphosphate (CTP), cytidine-5'-diphosphate (CDP), cytidine-5'-monophosphate (CMP), cytidine, fluvastatin (Flu), aminopterin (Ami), argatroban (Arg), bivalirudin (Biv), dabigatran (Dab), methotrexate (Met), pyrimethamine (Pyr), trimethoprim (Tri), norfloxacin (Nor), formic acid (FA), and ammonium acetate (NH_4Ac) were purchased from Sigma-Aldrich (Buchs, CH). Human Alpha Thrombin and recombinant human dihydrofolate reductase (DHFR) were purchased from Innovative-research and Abcam (Cambridge, UK). H_2O, methanol (MeOH) and acetonitrile (ACN) for LC-MS were purchased from Chemsolute (Huberlab, Aesch, CH). Safety Alert: methotrexate and aminopterin are chemotherapy agents and we recommend that they be handled in a fume hood.

OPP-ESI-MS. MS analyses were performed on a TripleTOF 5600 (Sciex, Concord, ON) integrated with a 3D-printed OPP. The outer tube of the OPP was connected to an LC pump (Shimadzu LC-30AD) and the inner tube was connected to the Turbo V ion source (Sciex). Experiments were conducted in positive ion mode with a TOF-MS experiment of *m/z* 800-2500 or 1500-4000 for thrombin. Ionization settings were as follows: GS1 = 80; GS2 = 45; curtain gas = 30; TEM = 300, 400 or 500 °C and ISVF = 5000 V. Data were acquired with Analyst software (version 1.6.2).

Supplementary Table and Figures

	10 mM NH_4AC	0.1% FA	MeOH (0.1% FA)	Temperature
Condition 1	100 %	0%	0%	300 °C
Condition 2	100 %	0%	0%	500 °C
Condition 3	50 %	50 %	0%	300 °C
Condition 4	100 %	0%	0%	400 °C
Condition 5	0 %	100 %	0%	400 °C
Condition 6	50%	50 %	0%	400 °C

Table S1. Conditions applied in screening assays of thrombin and human DHFR

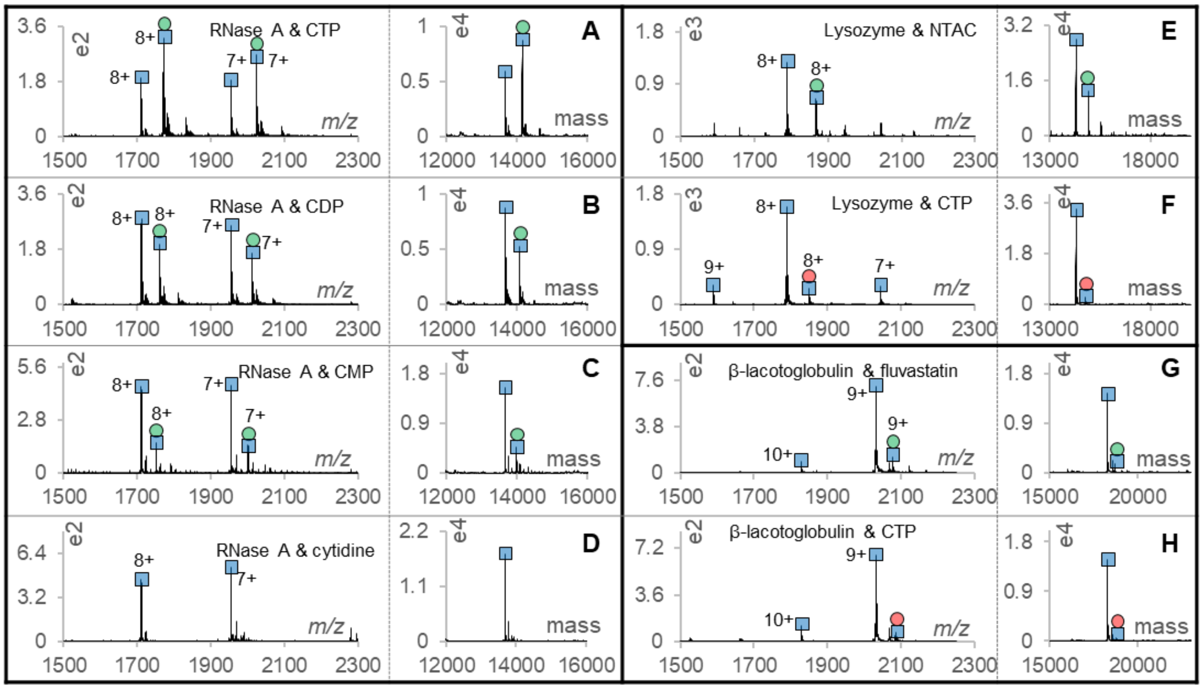

Figure S1. Probing PMIs under the constant native condition of 10 mM NH_4Ac. In each panel, the left part is the raw spectrum, and the right part is the corresponding reconstructed spectrum after deconvolution based on multiple charge states. The proteins and ligands are freshly mixed and incubated for 5 minutes, then dispensed dropwise onto OPP by a syringe. The investigations of RNase A (100 μM): **A** with CTP (1 mM), **B** with CDP (1 mM), **C** with CMP (1 mM), and **D** with cytidine (1 mM). The investigations of lysozyme (100 μM): **E** with NTAC (1 mM) and **F** with CTP (1 mM). The investigations of beta-lactoglobulin (50 μM): **G** with fluvastatin (500 μM) and **H** with CTP (500 μM). The blue square, green circle, and red circle indicate the unbound protein, the true-positive ligand, and the false-positive ligand respectively.

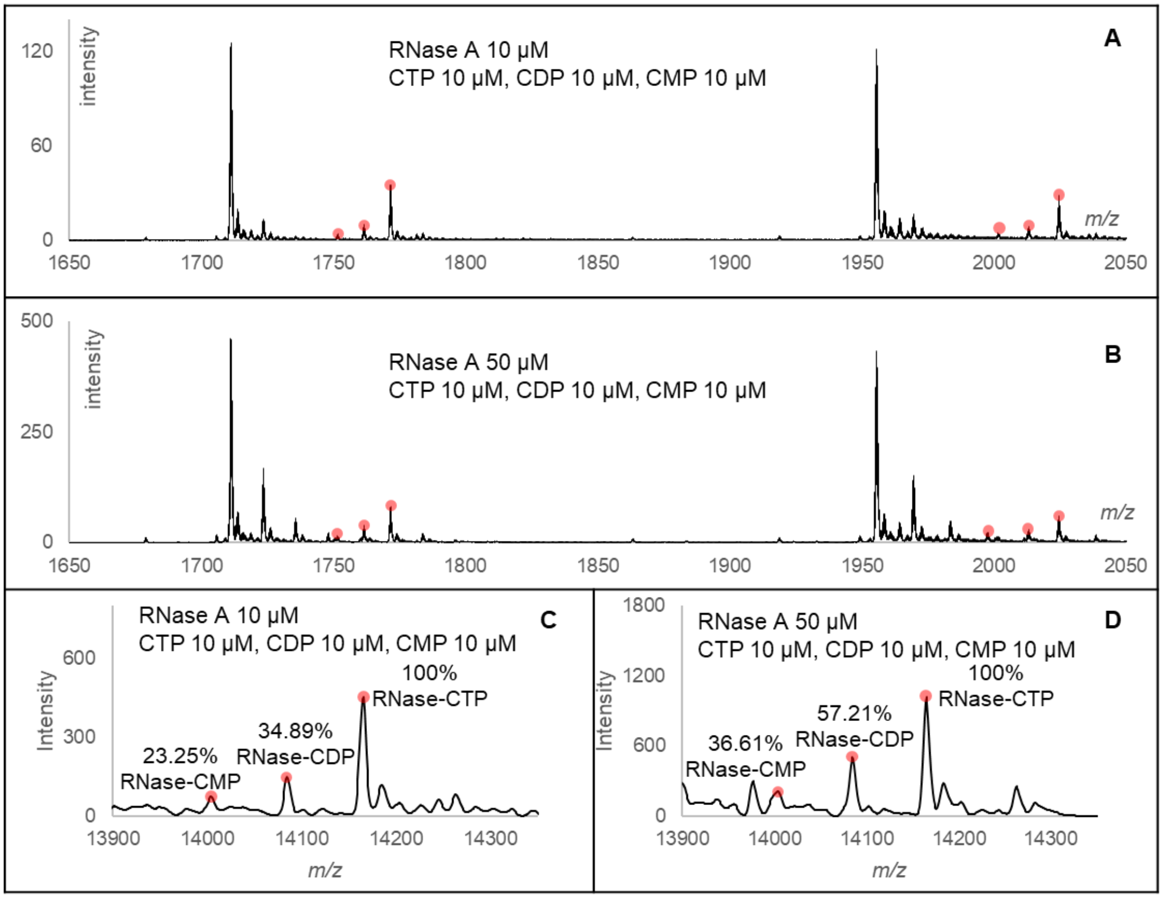

Figure S2. Ranking the affinity of CTP, CDP, and CMP to RNase A.

A. the raw spectrum of competitive experiments, in which three ligands have the same concentration of 10 μM and the concentration of RNase A is 10 μM, i.e. lower than the total concentration of ligands.

B. the raw spectrum of noncompetitive experiments, in which three ligands have the same concentration of 10 μM and the concentration of RNase A is 50 μM i.e. higher than the total concentration of ligands.

C. the reconstructed spectrum of competitive experiments after deconvolution.

D. the reconstructed spectrum of noncompetitive experiments after deconvolution.

C. (RNase A, μM)	C. (CTP, μM)	R ([PL]/[P])
5	0.5	0.0434
5	1	0.0628
5	2	0.0823
5	5	0.2776
5	10	0.5001
5	15	0.7850
5	20	0.7904
5	35	1.2023
5	50	2.2550

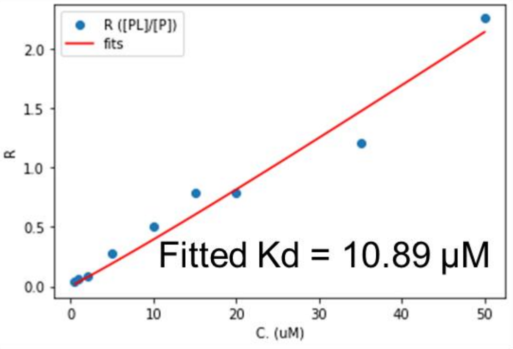

C. (RNase A, μM)	C. (CDP, μM)	R ([PL]/[P])
5	2	0.0280
5	5	0.0911
5	10	0.1233
5	15	0.1926
5	20	0.2866
5	35	0.5130
5	50	0.5463

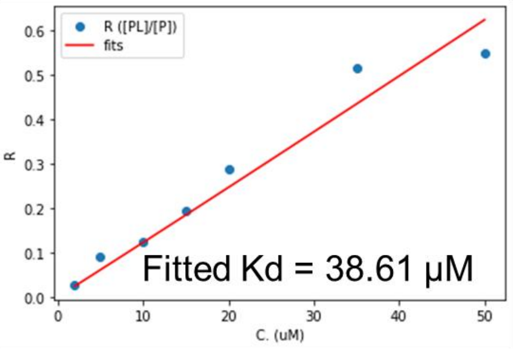

Measured R values versus the concentration of ligands (CTP/CDP) were fitted by the nonlinear least squares method to fit the function below.

$$R = \frac{-(K_d - 0.5 \cdot [CXP] + 0.5 \cdot [RNaseA]) + \sqrt{(K_d - 0.5 \cdot [CXP] + 0.5[RNaseA])^2 + 2 \cdot K_d \cdot [CXP]}}{2 \cdot K_d}$$

Figure S3. Measuring the Kd of CTP and CDP to RNase A by titration assays. The concentration of RNase A is constant at 5 μM. The ligands CTP and CDP cover a concentration range from 0.5 μM to 50 μM. For each ligand concentration, *R* is calculated by dividing the peak area of complex [PL] with the peak area of free protein [P]. The generated *R* values are plotted versus the concentrations of ligands. With the equation above, the points are fitted by the nonlinear least squares methods. The measured Kd is 10.89 μM for CTP and 38.61 μM for CDP.

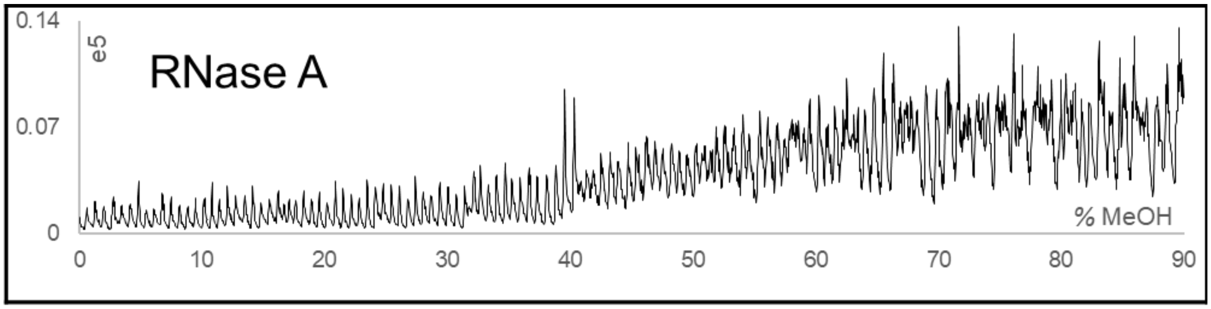

Figure S4. The BPCs of RNase A over the gradient from 0 to 90% MeOH.

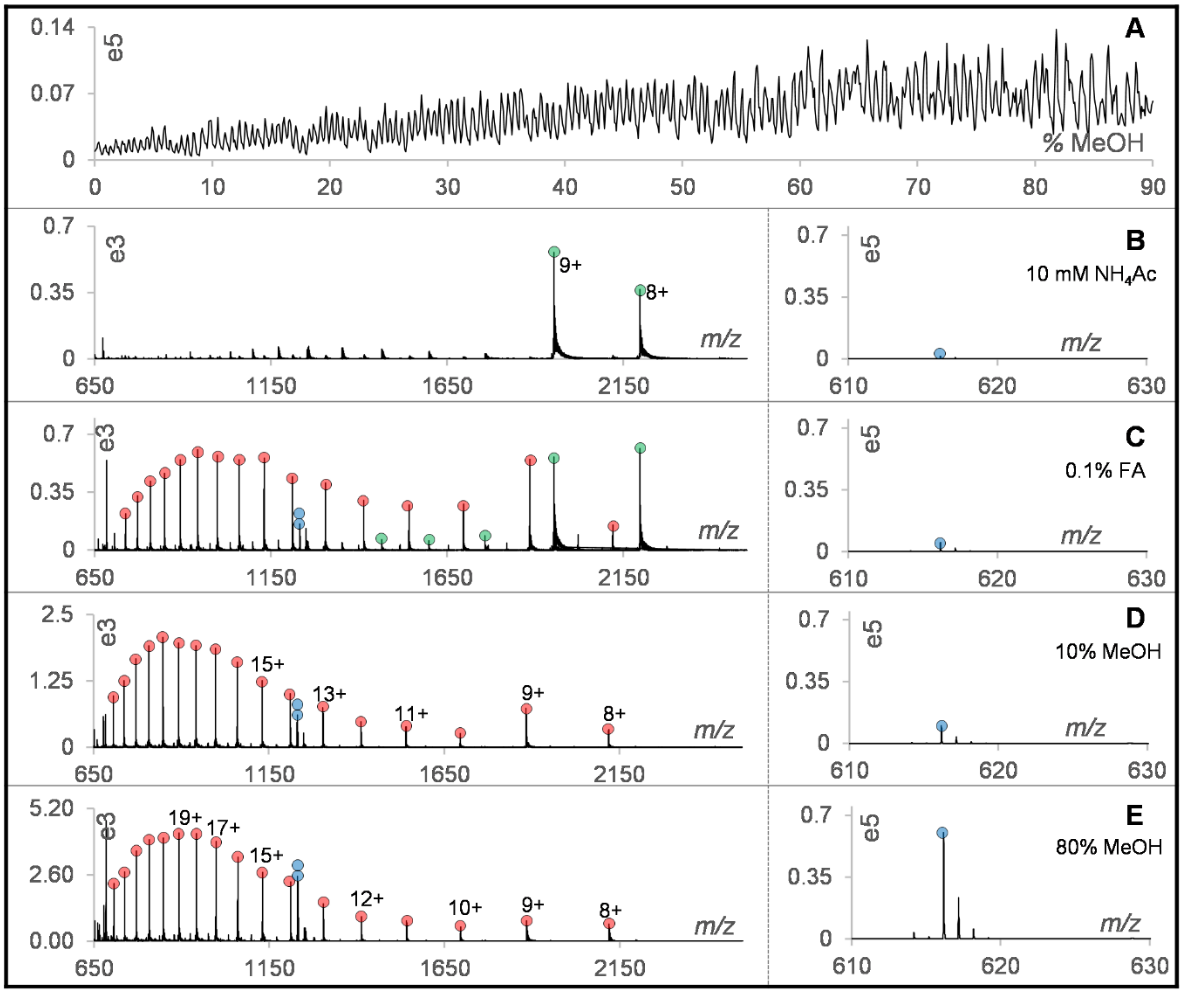

Figure S5. Modulating CSD of myoglobin by increasing the proportion of methanol in the OPP solvent. **A**. the BPC of the heme-bound myoglobin and/or the free myoglobin over the gradient from 0 to 90% MeOH. Raw spectra of the heme-bound myoglobin and/or the free myoglobin at various proportions of MeOH: **B**. 100% 10 mM NH_4Ac, **C**. 100% H_2O containing 0.1% formic acid, **D**. 10% MeOH, **E**. 80% MeOH. The green and red circles indicate the CSD of the heme-bound myoglobin and the free myoglobin respectively. The blue circle represents the peak of heme (*m/z* of 616.17) and the "double-blue circle" denotes the peak of $(2M+H)^+$ at *m/z* 1231.27. In panels **B** to **E**, the left part is the spectrum of the free myoglobin or the heme-bound myoglobin and the right part shows the heme (*m/z* of 616.17) with a constant y scale.

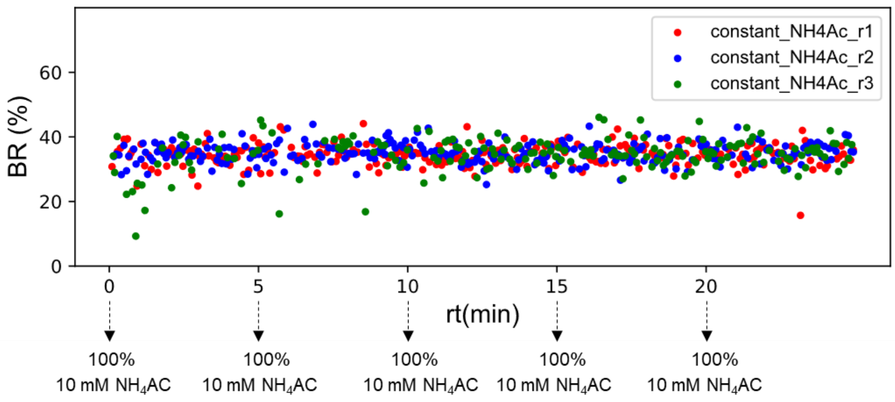

Figure S6. Reproducibility of the gOPP-ESI-MS approach under native condition. Triplicate analyses of CDP interacting with RNase A at constant 10 mM NH_4Ac for 25 min. Binding ratio, BR is the ratio [PL]/([PL] + [P]).

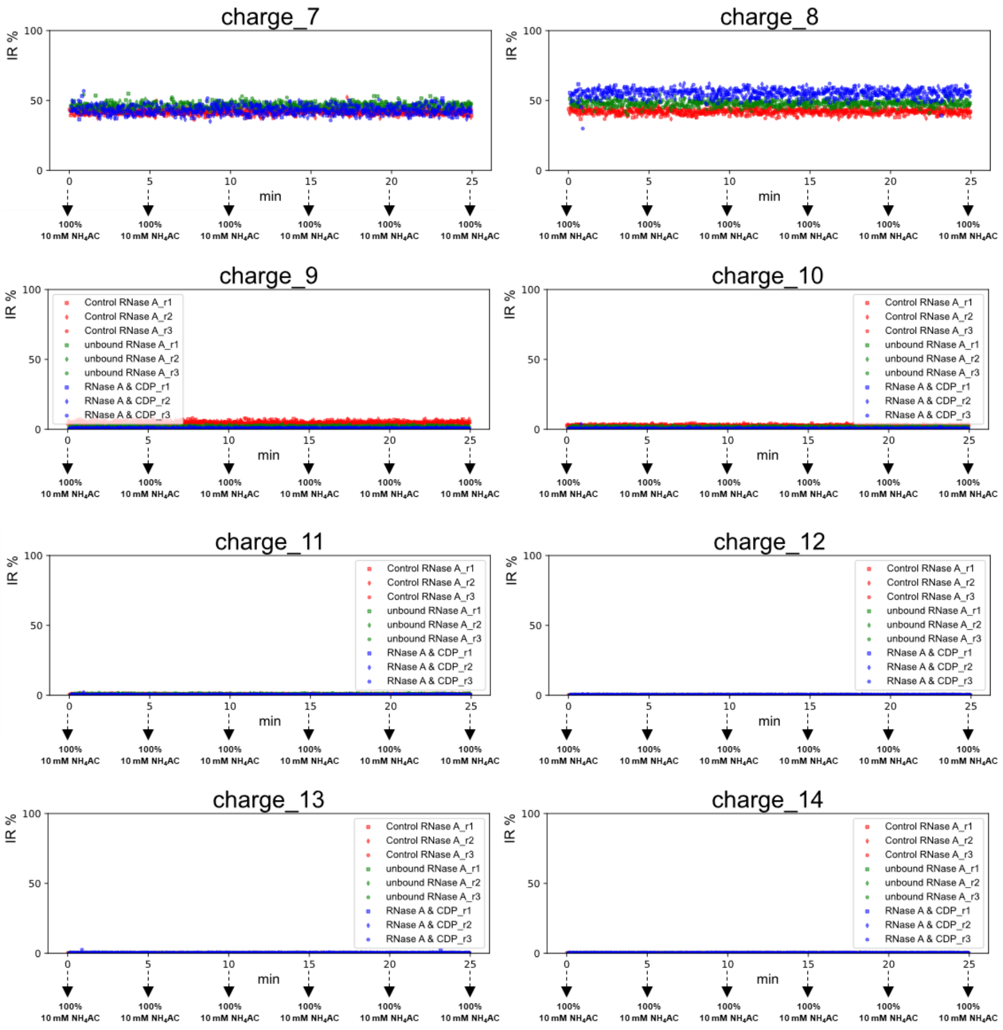

Figure S7. Triplicates analyses of the interaction between CDP and RNase A under the native conditions. The intensity ratios of all charges remain unchanged. There are two samples, the control sample containing RNase A without CDP and the experimental sample containing both RNase A and CDP. The RNase A in the control sample was named as "control-RNase A" and the free RNase A in the experimental sample was named as "unbound-RNase A". Charge ratios are dominated by charges 7+ and 8+.

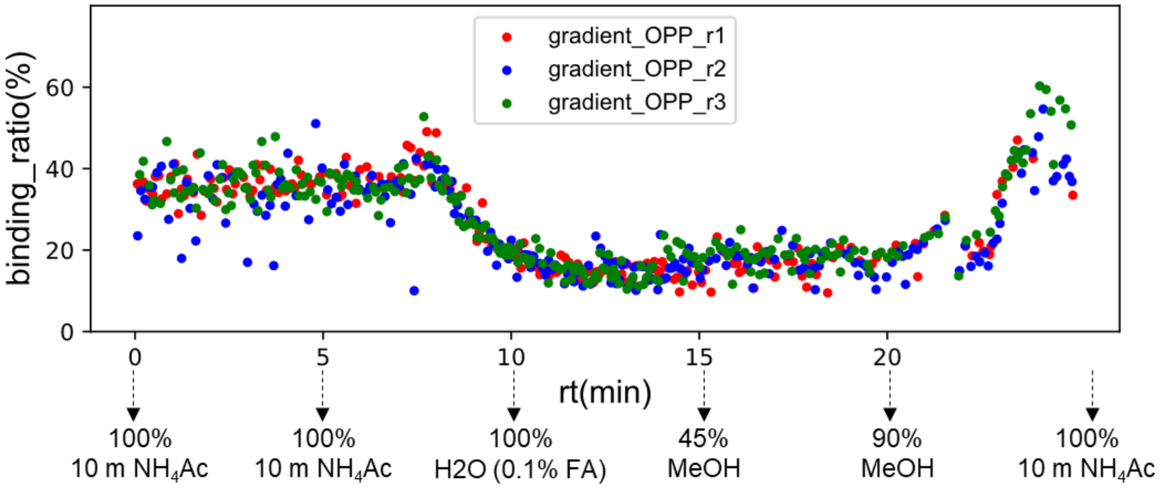

Figure S8. Reproducibility of the gOPP-ESI-MS approach under a gradient. A. triplicate analyses of CDP interacting with RNase A over a MeOH gradient. Binding ratio is [PL]/([PL] + [P]). Gradient: 0-5 min, constant at 100% 10 mM NH_4Ac, 5-10 min, change from 100% 10 mM NH_4Ac to 100% H_2O containing 0.1% FA, 10-20 min, change from 0 to 90% MeOH. 20-25 min, equilibrate at 100% 10 mM NH_4Ac.

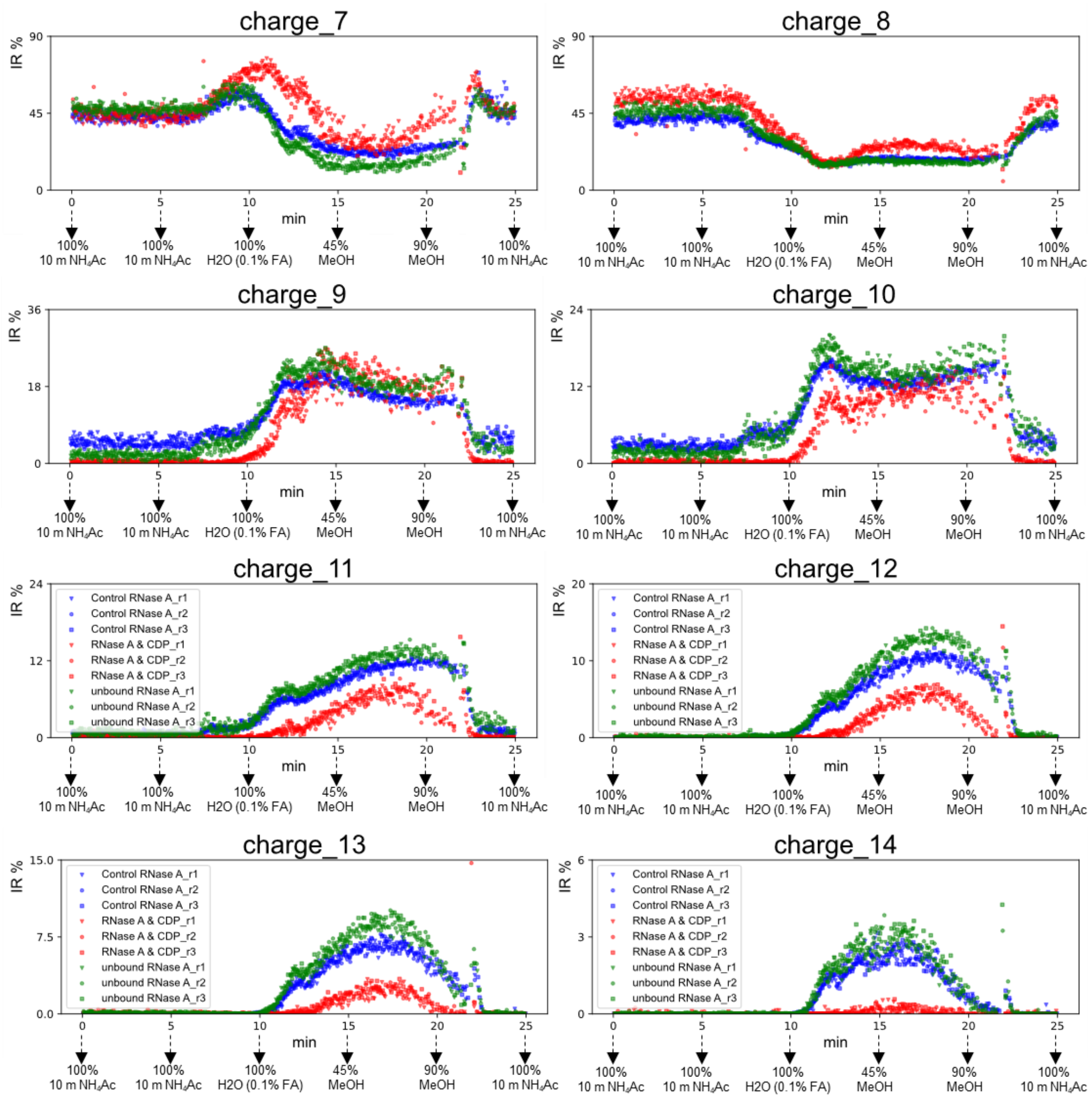

Figure S9. Triplicates analyses of the interaction between CDP and RNase A under a gradient, that consist of 0-5 min, constant at 100% 10 mM NH_4Ac, 5-10 min, change from 100% 10 mM NH_4Ac to 100% H_2O containing 0.1% formic acid, 10-20 min, change from 0 to 90% MeOH. 20-25 min, equilibrate at 100% 10 mM NH_4Ac. The spikes around 21 min may be caused by the sudden change from 90% MeOH to 100% 10 mM NH_4Ac. RNase A in the control sample is named as “control-RNase A”, free RNase A in the experimental sample was named as “unbound-RNase A” and the complex is "RNase A & CDP". The suffix "rx" indicates the replicate number.

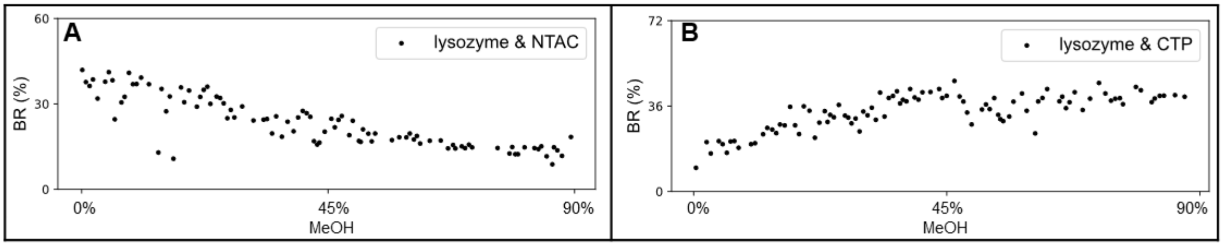

Figure S10. Binding ratio differentiations between the true PMIs and non-specific bindings. **A**. binding ratios of lysozyme and NTAC; **B**. binding ratios of lysozyme and CTP.

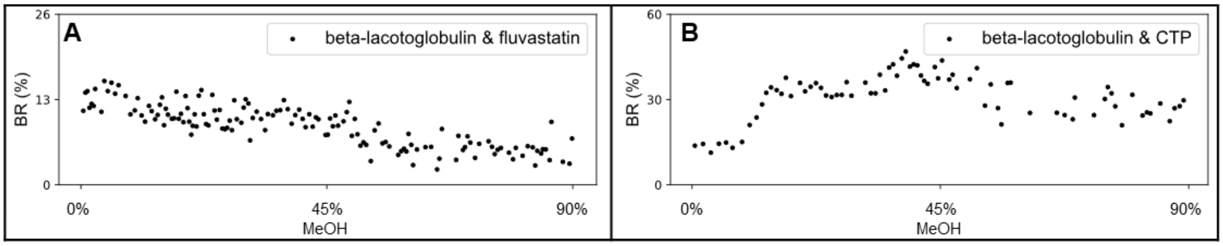

Figure S11. Binding ratio differentiations between the true PMIs and non-specific bindings. **A**. binding ratios of beta-lactoglobulin and fluvastatin; **B**. binding ratios of beta-lactoglobulin and CTP.

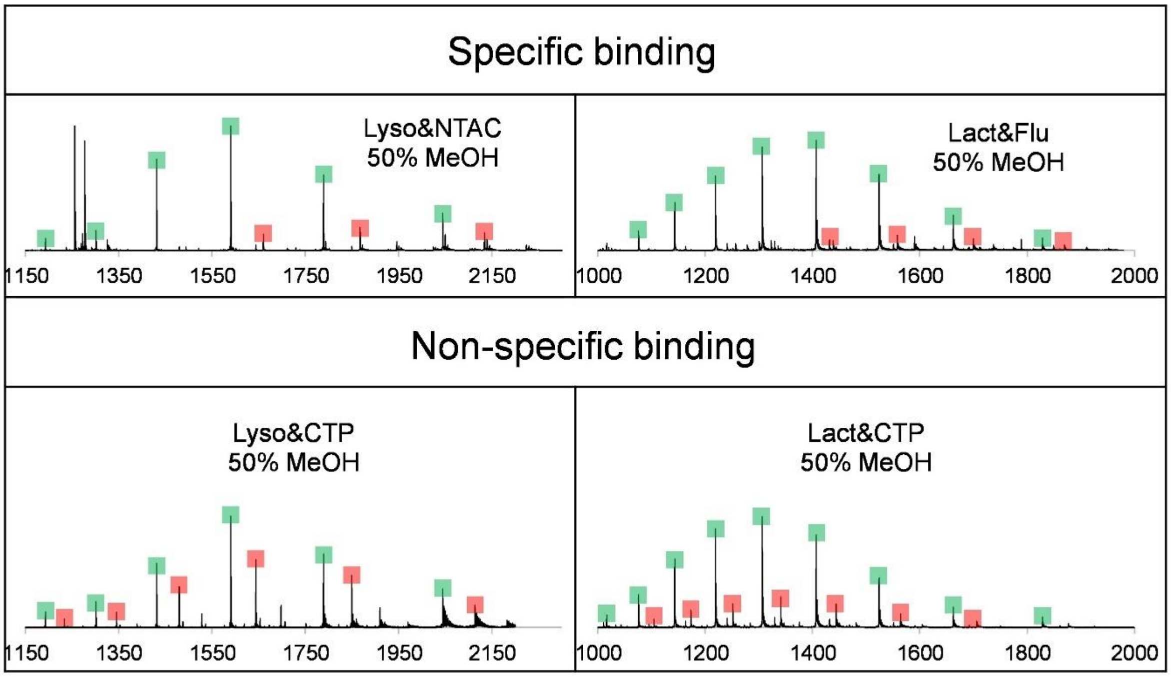

Figure S12. Differentiations between the true PMIs and non-specific bindings.

Raw spectra of NTAC-bound lysozyme, CTP-bound lysozyme, fluvastatin-bound beta-lactoglobulin and CTP-bound beta-lactoglobulin. The green squares represent the protein and the red squares the protein & ligand complex.

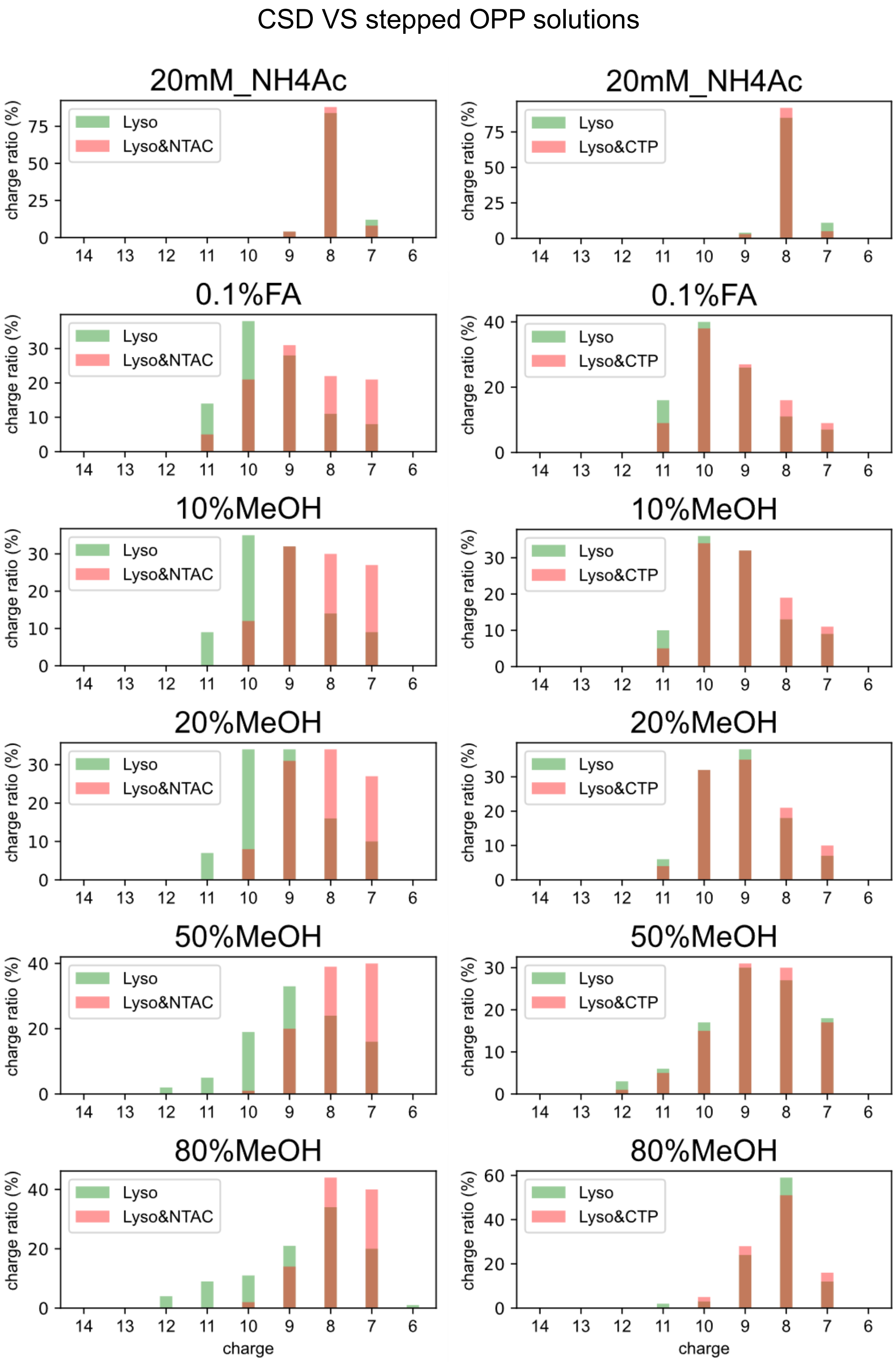

Figure S13. CSD comparation (lysozyme) between the true PMIs (lysozyme-NTAC) and nonspecific binding (lysozyme-CTP) under stepped OPP solutions.

CSD VS stepped OPP solutions

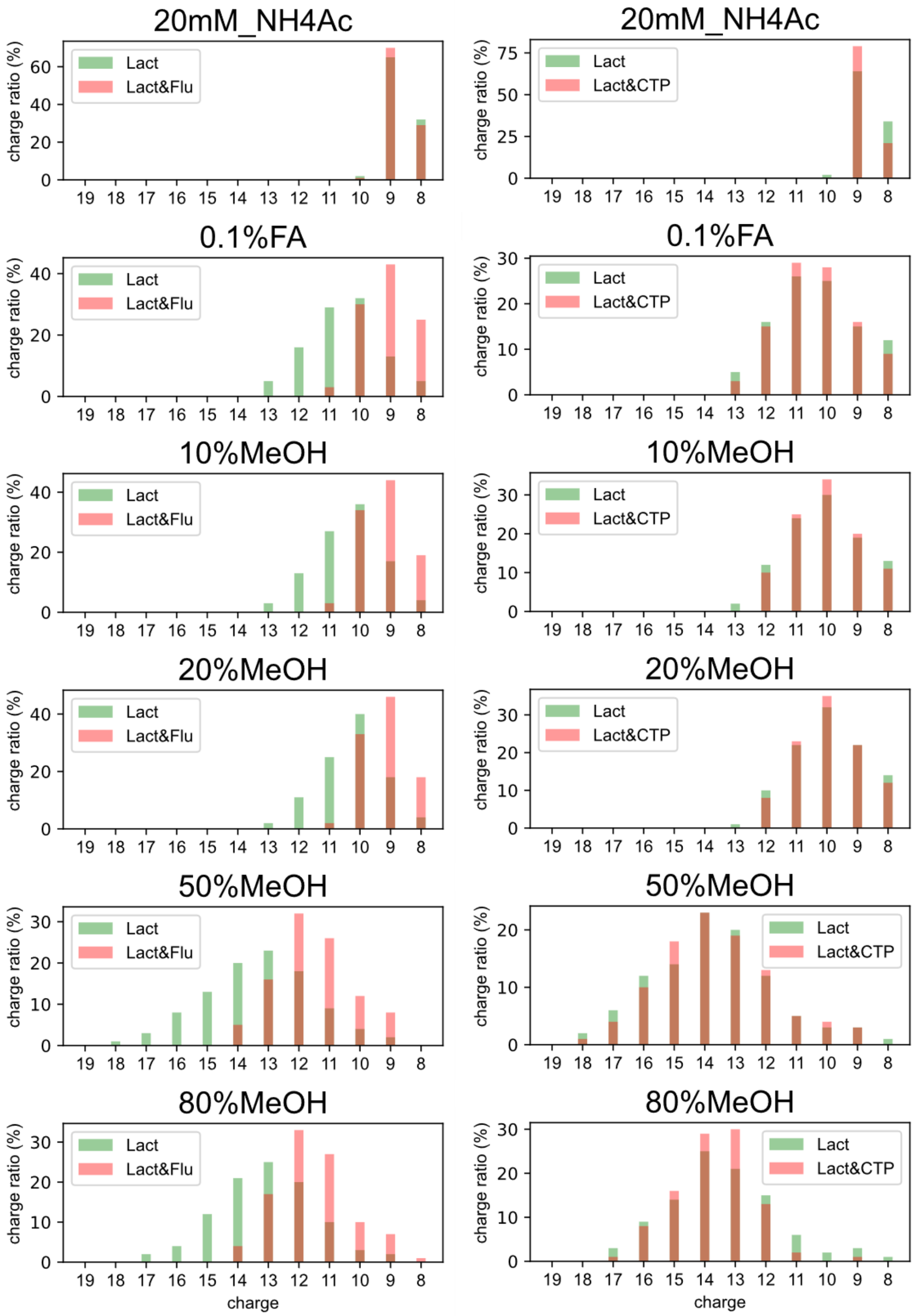

Figure S14. CSD comparation (beta-lactoglobulin) between the true PMIs (beta-lactoglobulin-fluvastatin) and nonspecific binding (beta-lactoglobulin-CTP) under stepped OPP solutions.

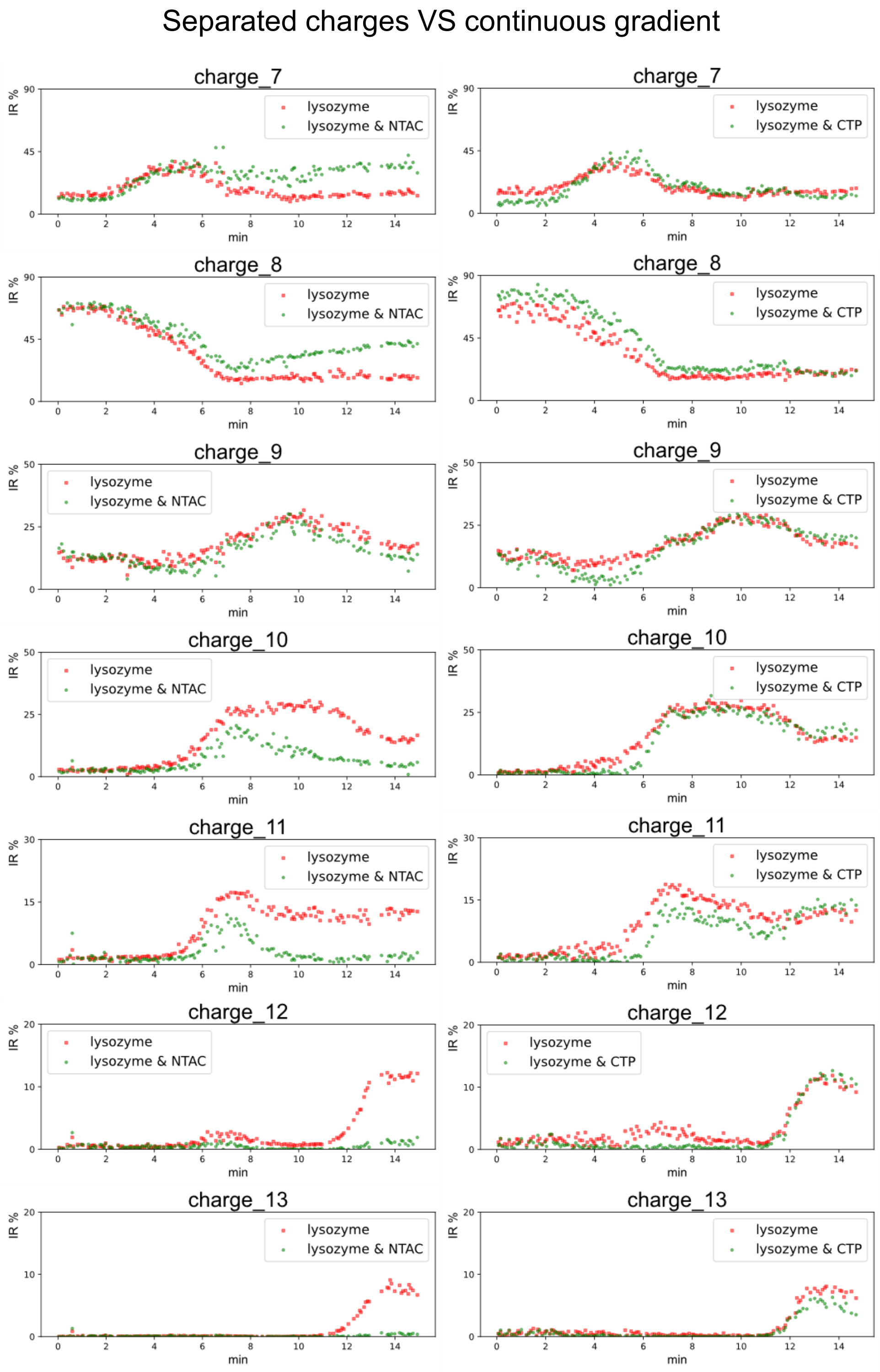

Figure S15. Comparation between the true PMIs (lysozyme-NTAC) and nonspecific binding (lysozyme-CTP) in CSD over the gradient. Gradient: 0-5 min, change from 100% 10 mM NH_4Ac to 100% H_2O containing 0.1% formic acid, 5-15 min, change from 0 to 90% MeOH.

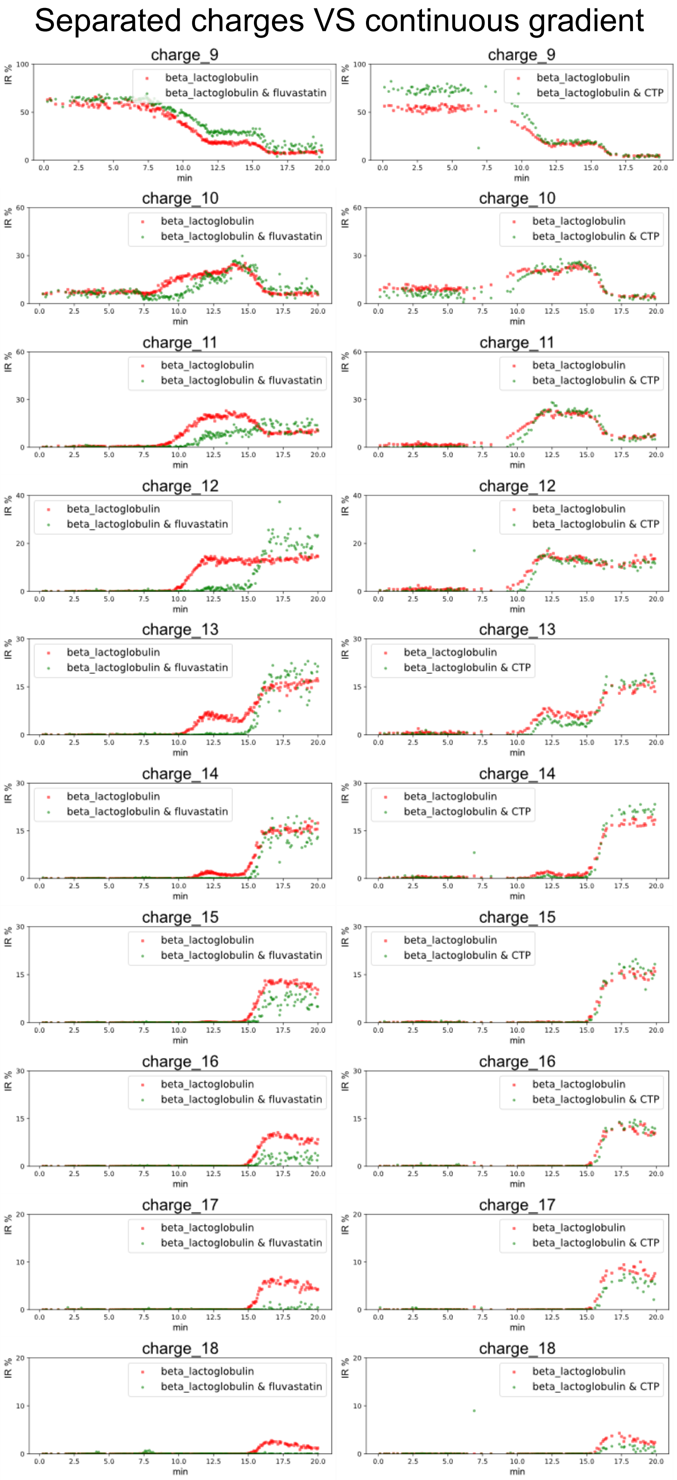

Figure S16. Comparation between the true PMIs (beta-lactoglobulin-fluvastatin) and nonspecific binding (beta-lactoglobulin-CTP) in CSD over the gradient. Gradient: 0-5 min, constant at 100% 10 mM NH_4Ac, 5-10 min, change from 100% 10 mM NH_4Ac to 100% H_2O containing 0.1% formic acid, 10-20 min, change from 0 to 90% MeOH.

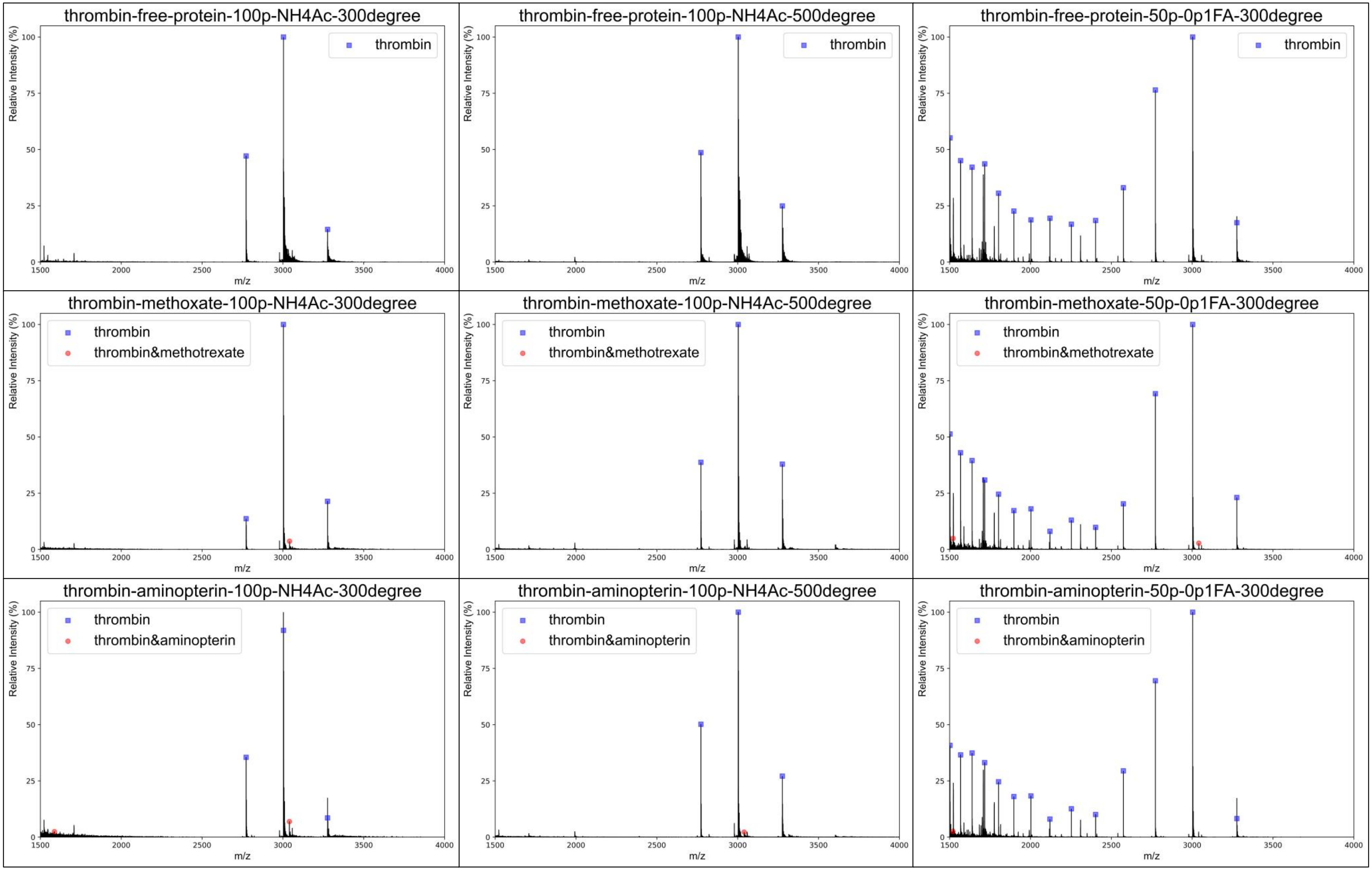
thrombin-free-protein-100p-NH4Ac-300degree
thrombin-free-protein-100p-NH4Ac-500degree
thrombin-free-protein-50p-0p1FA-300degree
thrombin-methoxate-100p-NH4Ac-300degree
thrombin-methoxate-100p-NH4Ac-500degree
thrombin-methoxate-50p-0p1FA-300degree
thrombin-aminopterin-100p-NH4Ac-300degree
thrombin-aminopterin-100p-NH4Ac-500degree
thrombin-aminopterin-50p-0p1FA-300degree
thrombin
thrombin&methotrexate
thrombin&aminopterin
Relative Intensity (%)
m/z

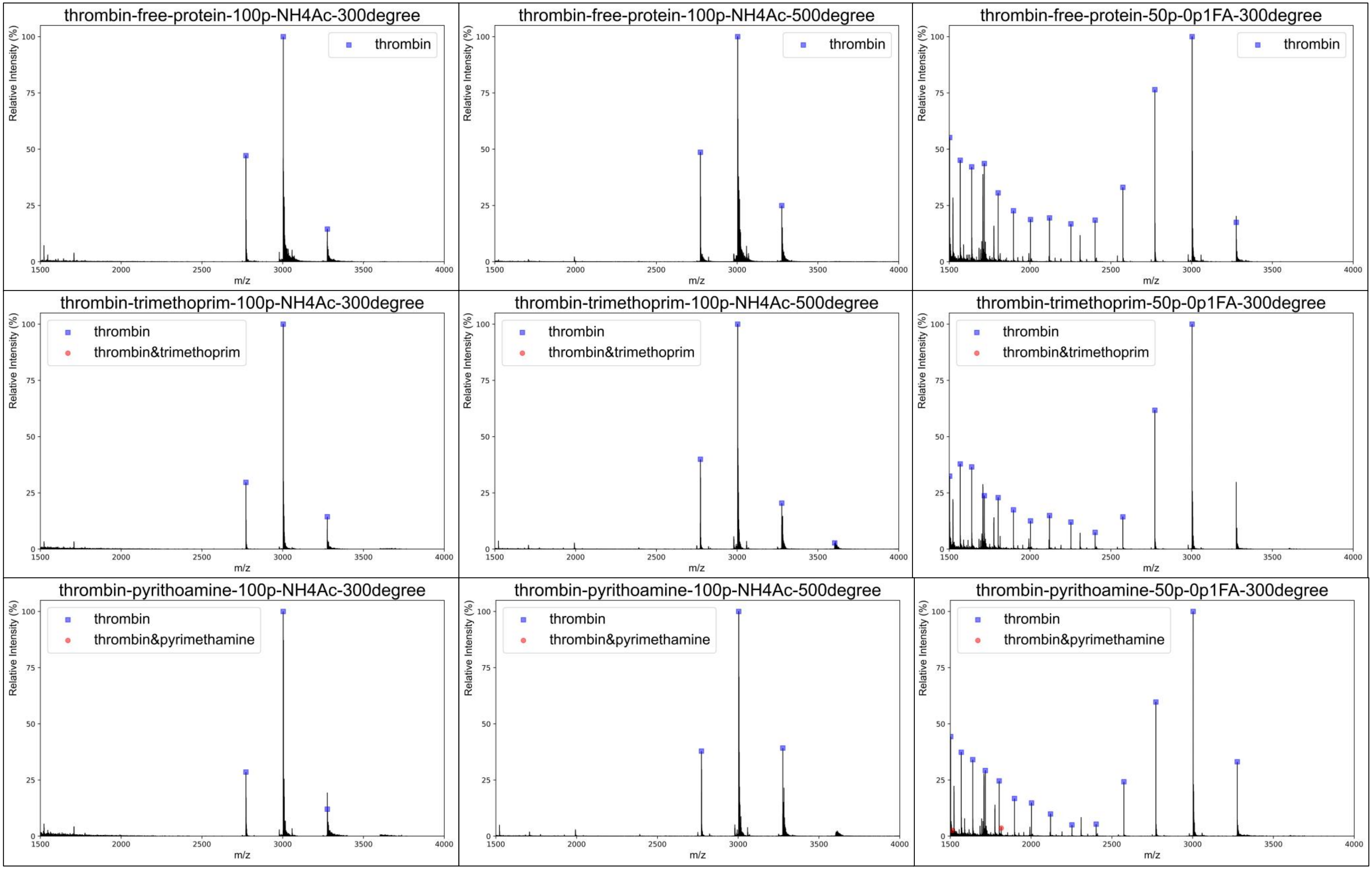

thrombin-free-protein-100p-NH4Ac-300degree
thrombin-free-protein-100p-NH4Ac-500degree
thrombin-free-protein-50p-0p1FA-300degree
thrombin-trimethoprim-100p-NH4Ac-300degree
thrombin-trimethoprim-100p-NH4Ac-500degree
thrombin-trimethoprim-50p-0p1FA-300degree
thrombin-pyrithoamine-100p-NH4Ac-300degree
thrombin-pyrithoamine-100p-NH4Ac-500degree
thrombin-pyrithoamine-50p-0p1FA-300degree
thrombin
thrombin&trimethoprim
thrombin&pyrimethamine
Relative Intensity (%)
m/z
0
25
50
75
100
1500
2000
2500
3000
3500
4000

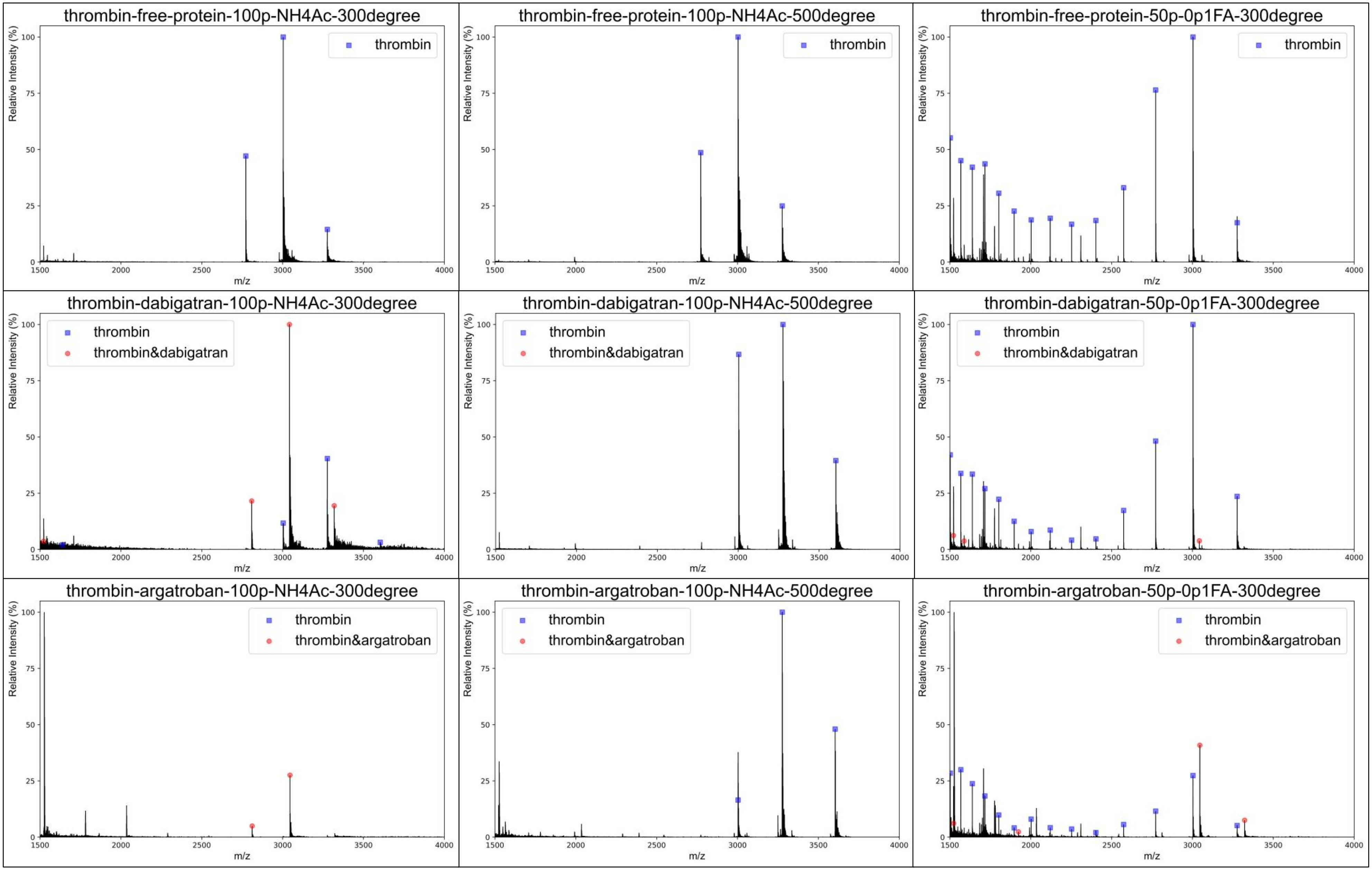
thrombin-free-protein-100p-NH4Ac-300degree
thrombin-free-protein-100p-NH4Ac-500degree
thrombin-free-protein-50p-0p1FA-300degree
thrombin-dabigatran-100p-NH4Ac-300degree
thrombin-dabigatran-100p-NH4Ac-500degree
thrombin-dabigatran-50p-0p1FA-300degree
thrombin-argatroban-100p-NH4Ac-300degree
thrombin-argatroban-100p-NH4Ac-500degree
thrombin-argatroban-50p-0p1FA-300degree
thrombin
thrombin&dabigatran
thrombin&argatroban
Relative Intensity (%)
m/z

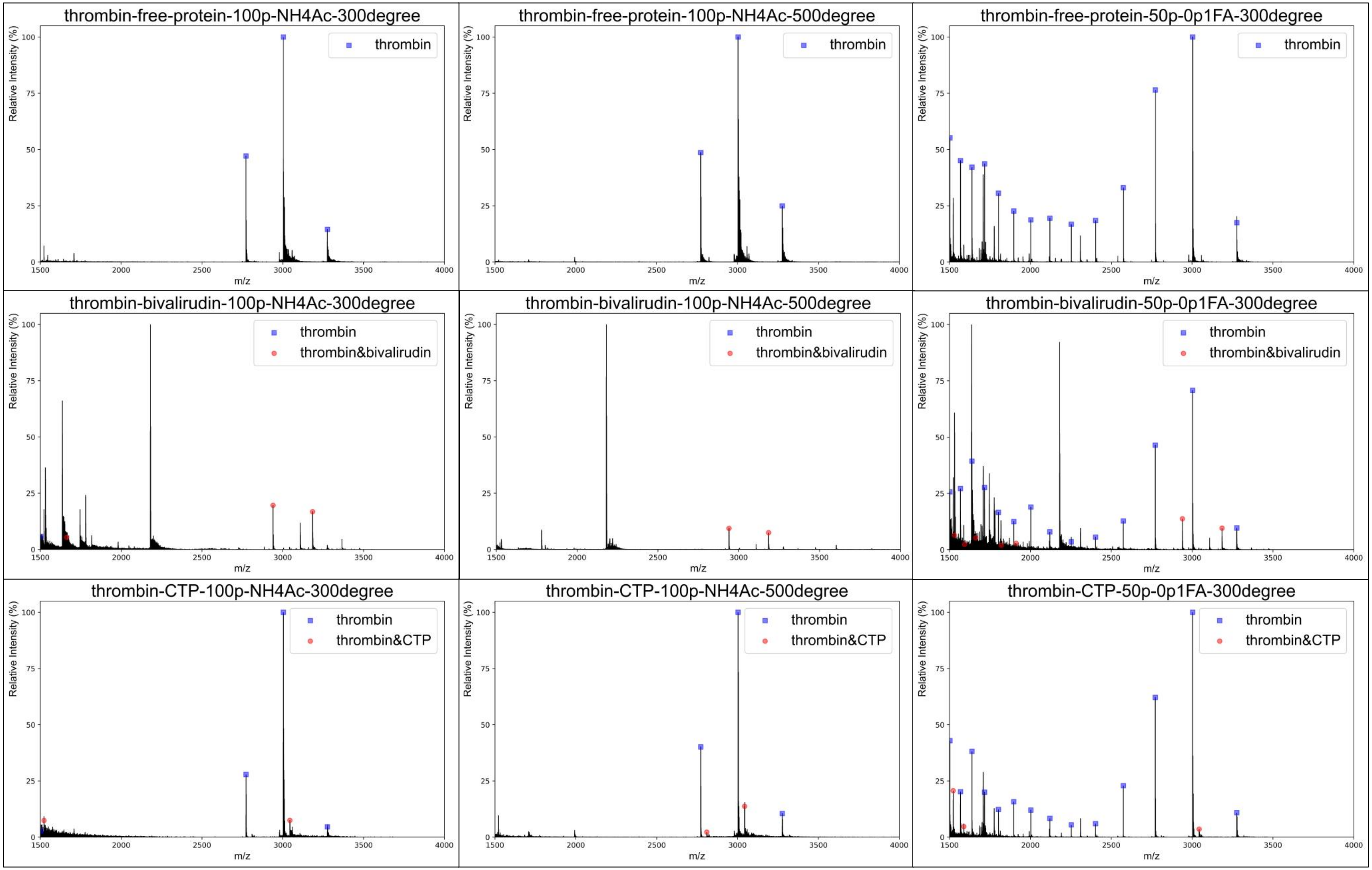
thrombin-free-protein-100p-NH4Ac-300degree
thrombin-free-protein-100p-NH4Ac-500degree
thrombin-free-protein-50p-0p1FA-300degree
thrombin-bivalirudin-100p-NH4Ac-300degree
thrombin-bivalirudin-100p-NH4Ac-500degree
thrombin-bivalirudin-50p-0p1FA-300degree
thrombin-CTP-100p-NH4Ac-300degree
thrombin-CTP-100p-NH4Ac-500degree
thrombin-CTP-50p-0p1FA-300degree
thrombin
thrombin&bivalirudin
thrombin&CTP
Relative Intensity (%)
m/z

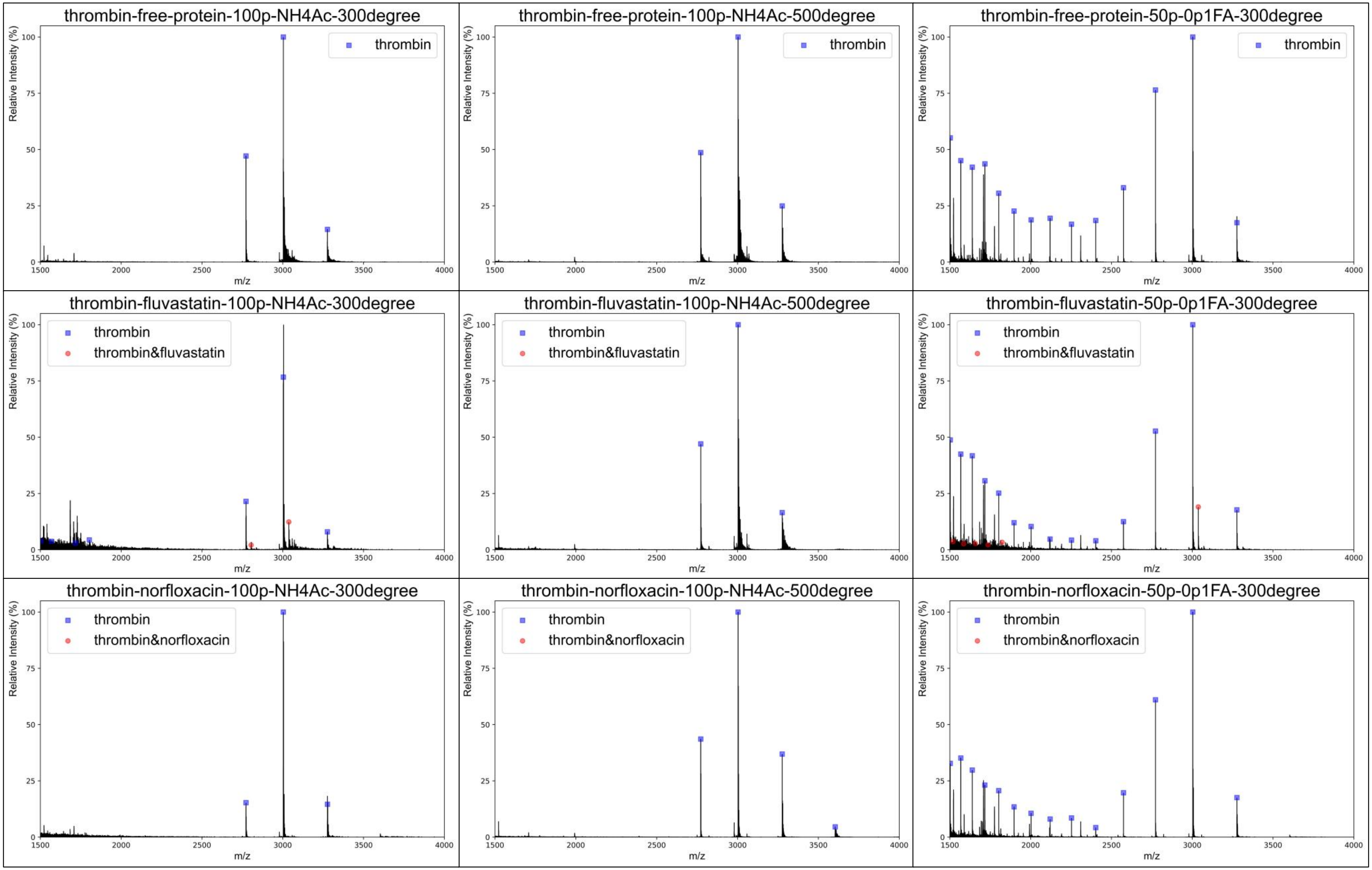
thrombin-free-protein-100p-NH4Ac-300degree
thrombin-free-protein-100p-NH4Ac-500degree
thrombin-free-protein-50p-0p1FA-300degree
thrombin-fluvastatin-100p-NH4Ac-300degree
thrombin-fluvastatin-100p-NH4Ac-500degree
thrombin-fluvastatin-50p-0p1FA-300degree
thrombin-norfloxacin-100p-NH4Ac-300degree
thrombin-norfloxacin-100p-NH4Ac-500degree
thrombin-norfloxacin-50p-0p1FA-300degree
thrombin
thrombin&fluvastatin
thrombin&norfloxacin
Relative Intensity (%)
m/z
0
25
50
75
100
1500
2000
2500
3000
3500
4000

Figure S17. Raw mass spectra of thrombin incubated with 10 ligands under three conditions. Condition 1: 100% 10 mM NH_4Ac & 300 °C, condition 2: 100% 10 mM NH_4Ac & 500 °C and condition 3: 50% 10 mM NH_4Ac & 50% H_2O containing 0.1% FA & 300 °C. 10 ligands include fluvastatin (Flu), aminopterin (Ami), argatroban (Arg), bivalirudin (Biv), dabigatran (Dab), methotrexate (Met), pyrimethamine (Pyr), trimethoprim (Tri), norfloxacin (Nor), cytidine-5'-triphosphate (CTP).

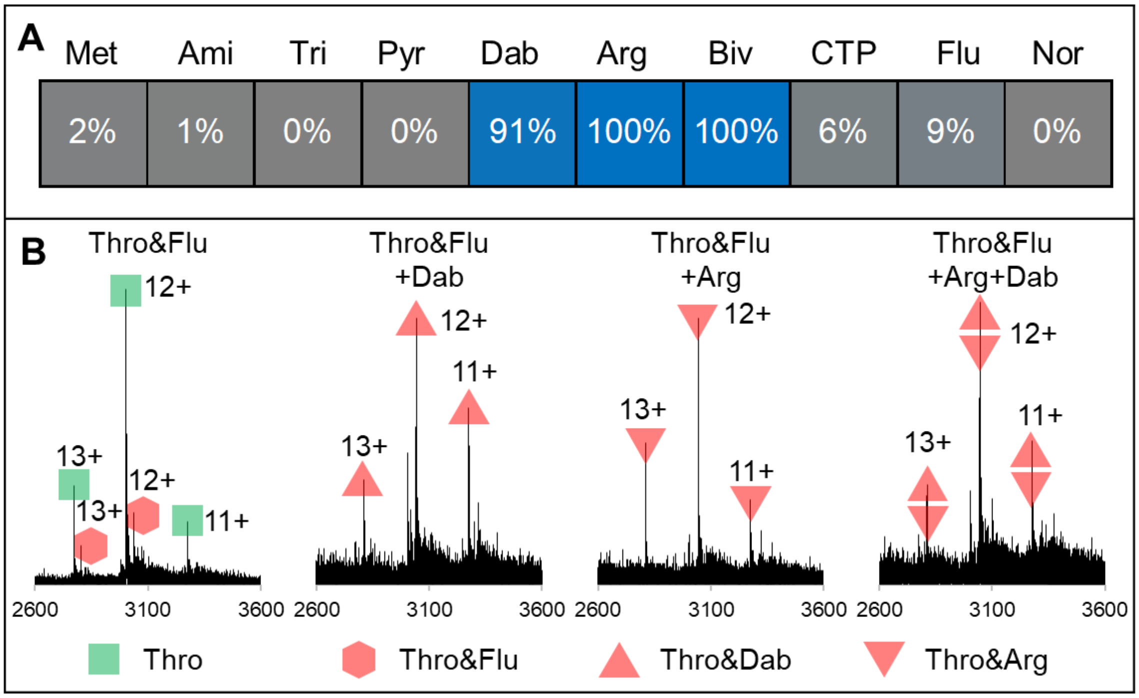

Figure S18. Screening thrombin and competition experiments under conditions 1: 100% 10 mM NH_4Ac & 300 °C. **A**. binding ratios with 10 ligands under condition 1. **B**. competition binding assays of thrombin & fluvastatin against dabigatran and argatroban under condition 1. Fluvastatin was incubated with thrombin for 5 minutes, followed by further adding dabigatran and argatroban individually and together.

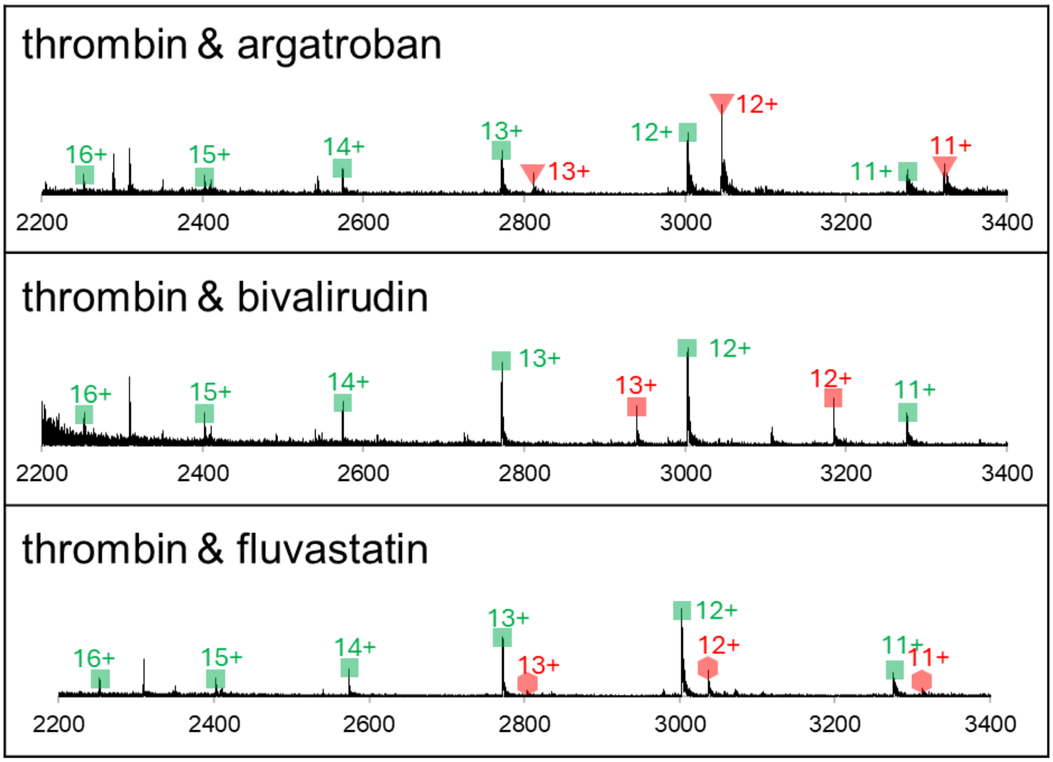

Figure S19. Raw mass spectra of thrombin & argatroban, thrombin & bivalirudin, and thrombin & fluvastatin of screenings under condition 3: 50% 10 mM NH_4Ac-50% H_2O containing 0.1% FA at 300 °C.

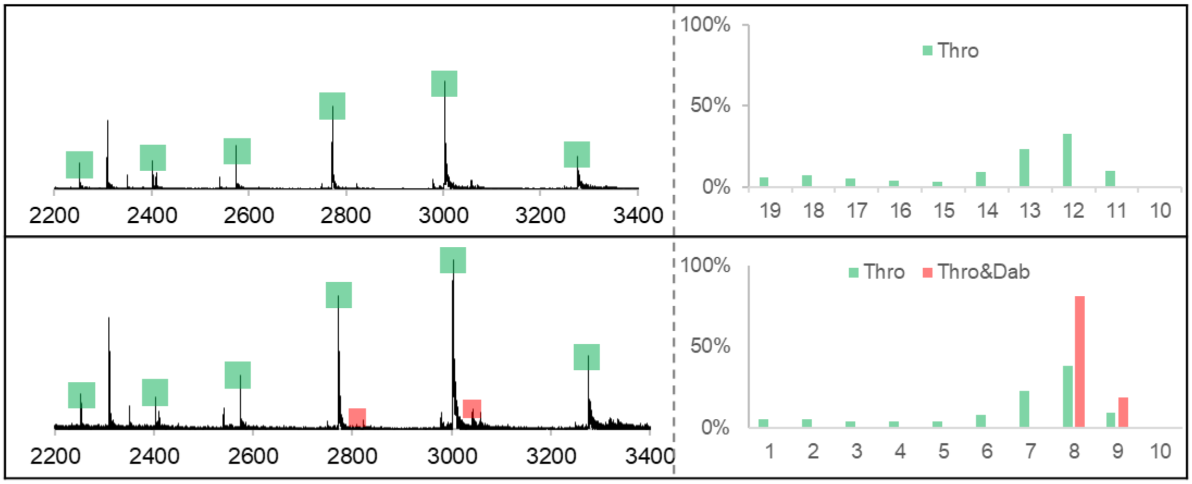

Figure S20. Raw mass spectra and CSD of free thrombin and the complex of thrombin & dabigatran under condition 3: 50% 10 mM NH_4Ac-50% H_2O containing 0.1% FA at 300 °C. The right part is corresponding CSD, x and y axes represent the charge states and the ratio of charge intensity.

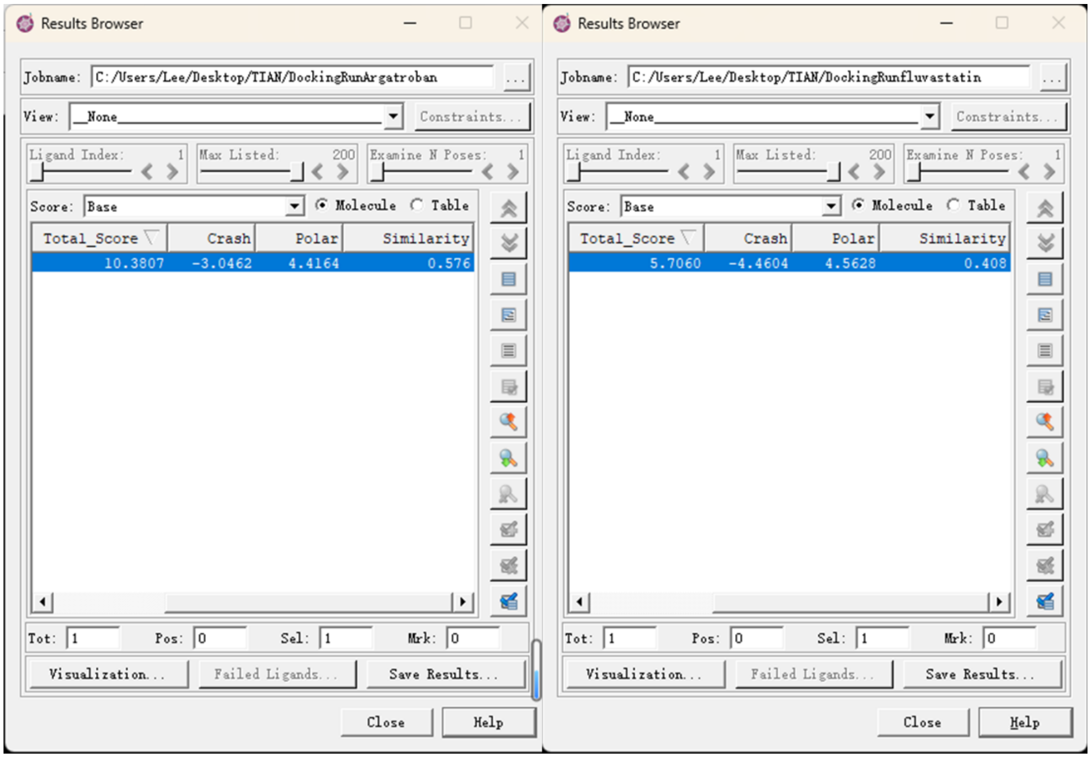

	Total Score	Crash	Polar	Similarity
Argatroban	10.3807	-3.0462	4.4164	0.576
Fluvastatin	5.7060	-4.4604	4.5628	0.408

Figure S21. Original outputs of dockings of argatroban versus fluvastatin. Docking was performed in SYBYL-X 2.0 and images depicted by PyMOL software.

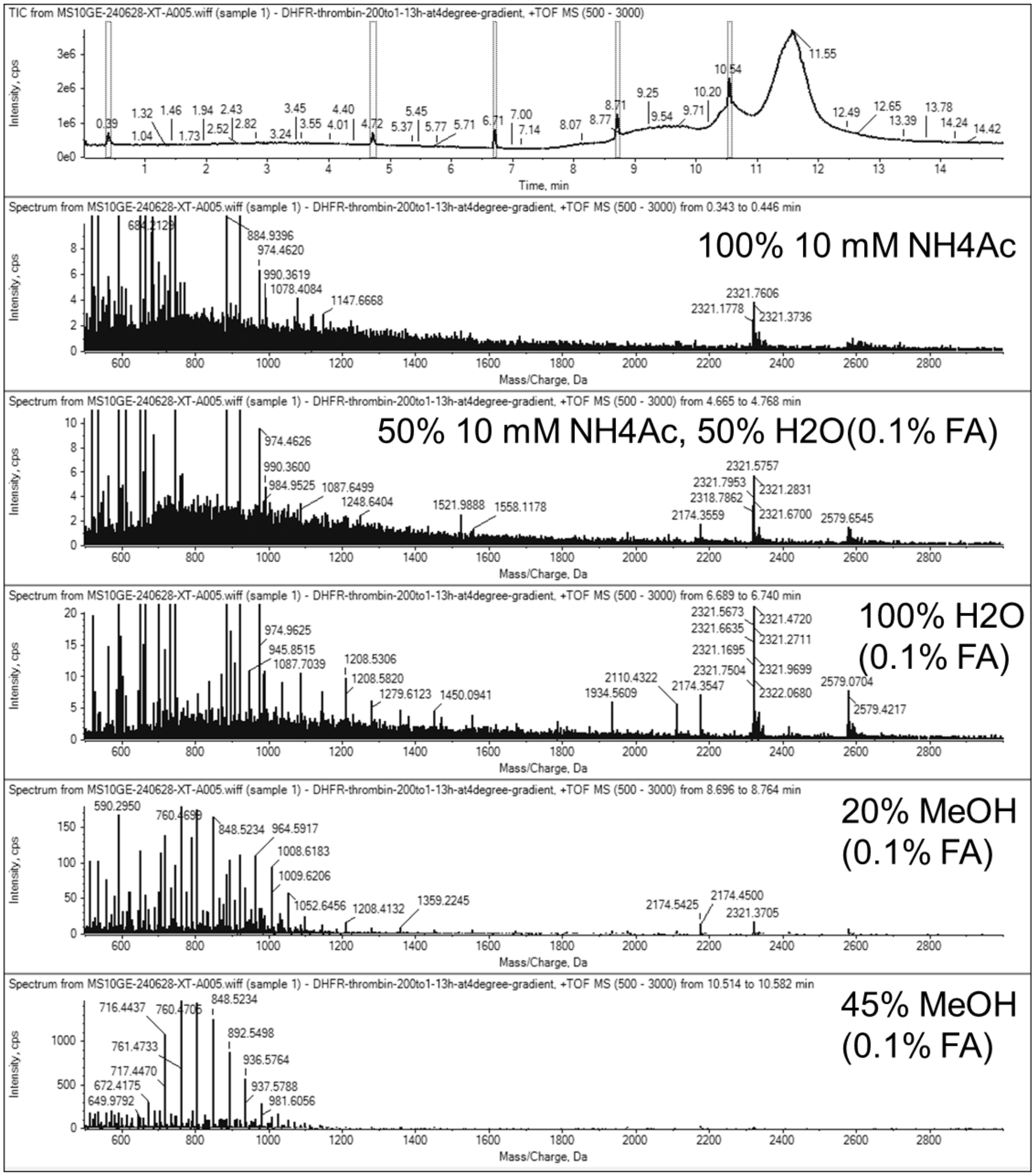

Sample:
free dehis-DHFR

Gradient:
0 min 100% 10 mM NH4Ac
5 min 100% H2O(0.1% FA)
10 min 50% MeOH(0.1% FA)
~2 min gradient delay

Figure S22. Rapid assessment of OPP solutions with a gradient. 1 uL of DHFR protein sample was manually pipetted onto the OPP at specific time points. It revealed the 100% H_2O containing 0.1% FA efficiently unfolded DHFR.

Met	Ami	Tri	Pyr	Dab	Arg	Biv	CTP	Flu	Nor
100%	100%	0%	0%	0%	NA	NA	0%	0%	0%

Figure S23. DHFR binding ratios of screenings against 10 ligands under condition 4: 100% 10 mM NH_4Ac & 400 °C. "NA" indicates no protein signal was observed in the measurement.

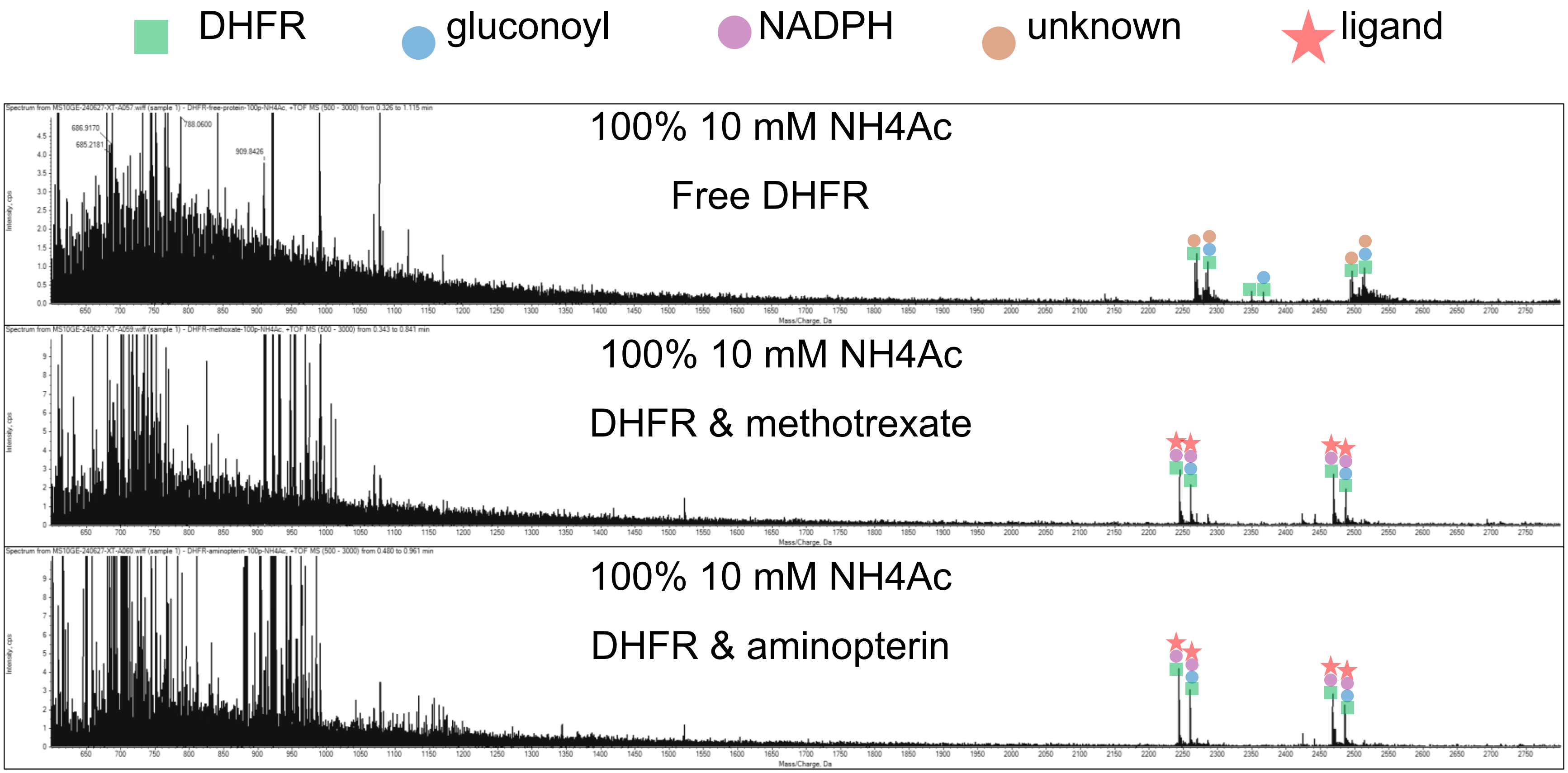
DHFR
gluconoyl
NADPH
unknown
ligand
100% 10 mM NH4Ac
Free DHFR
100% 10 mM NH4Ac
DHFR & methotrexate
100% 10 mM NH4Ac
DHFR & aminopterin

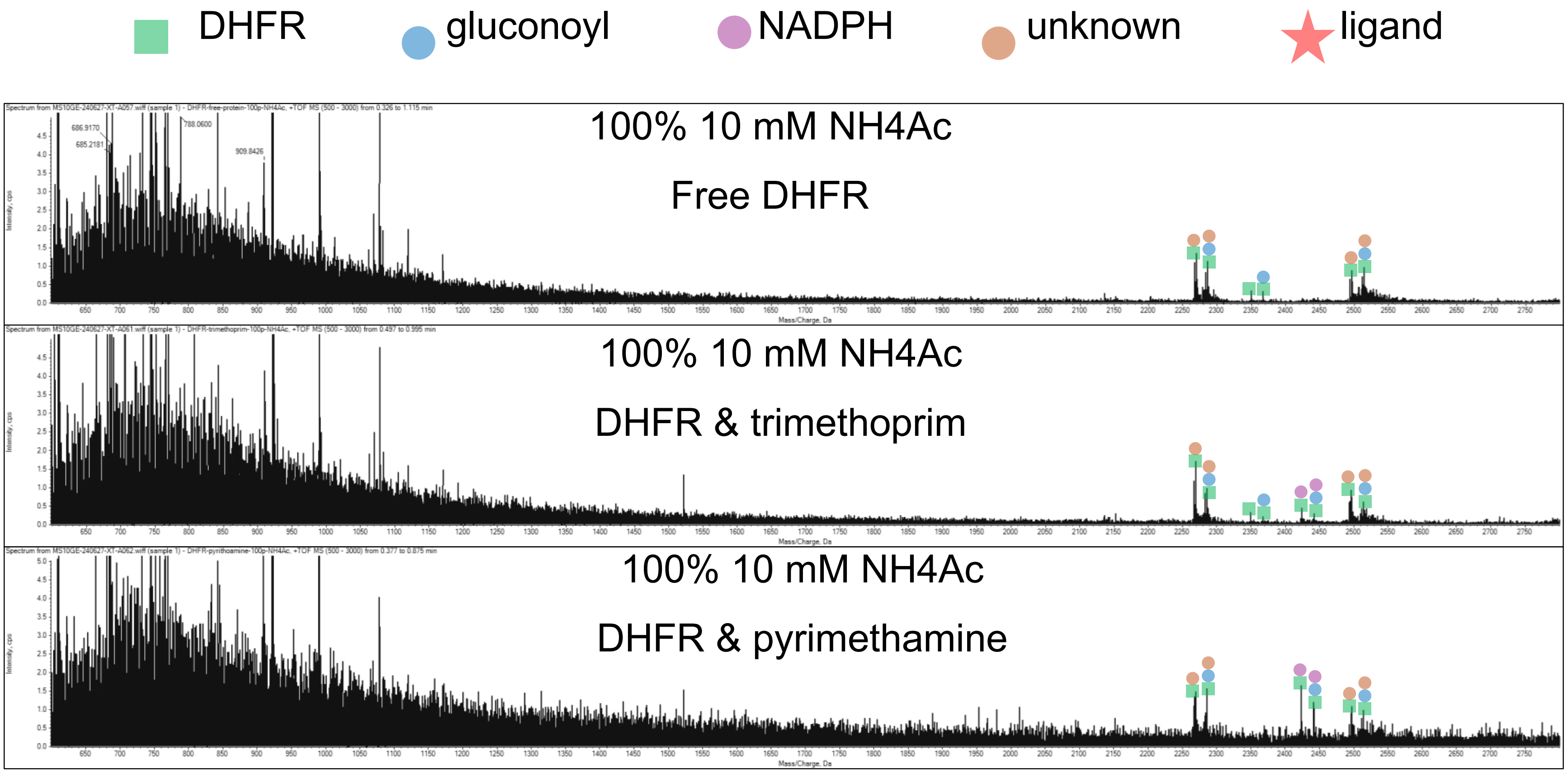
DHFR
gluconoyl
NADPH
unknown
ligand
100% 10 mM NH4Ac
Free DHFR
100% 10 mM NH4Ac
DHFR & trimethoprim
100% 10 mM NH4Ac
DHFR & pyrimethamine

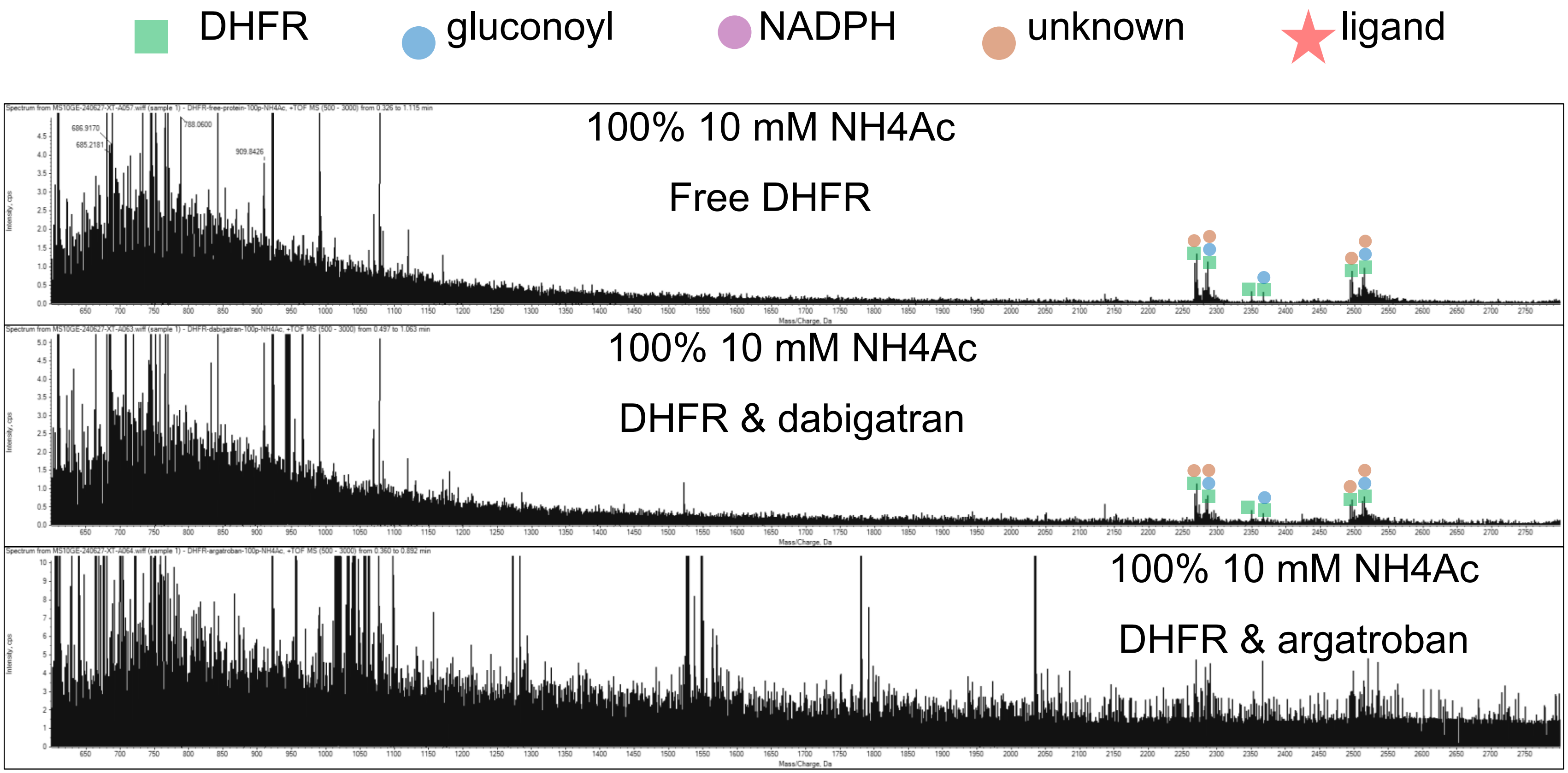
DHFR
gluconoyl
NADPH
unknown
ligand
100% 10 mM NH4Ac
Free DHFR
100% 10 mM NH4Ac
DHFR & dabigatran
100% 10 mM NH4Ac
DHFR & argatroban

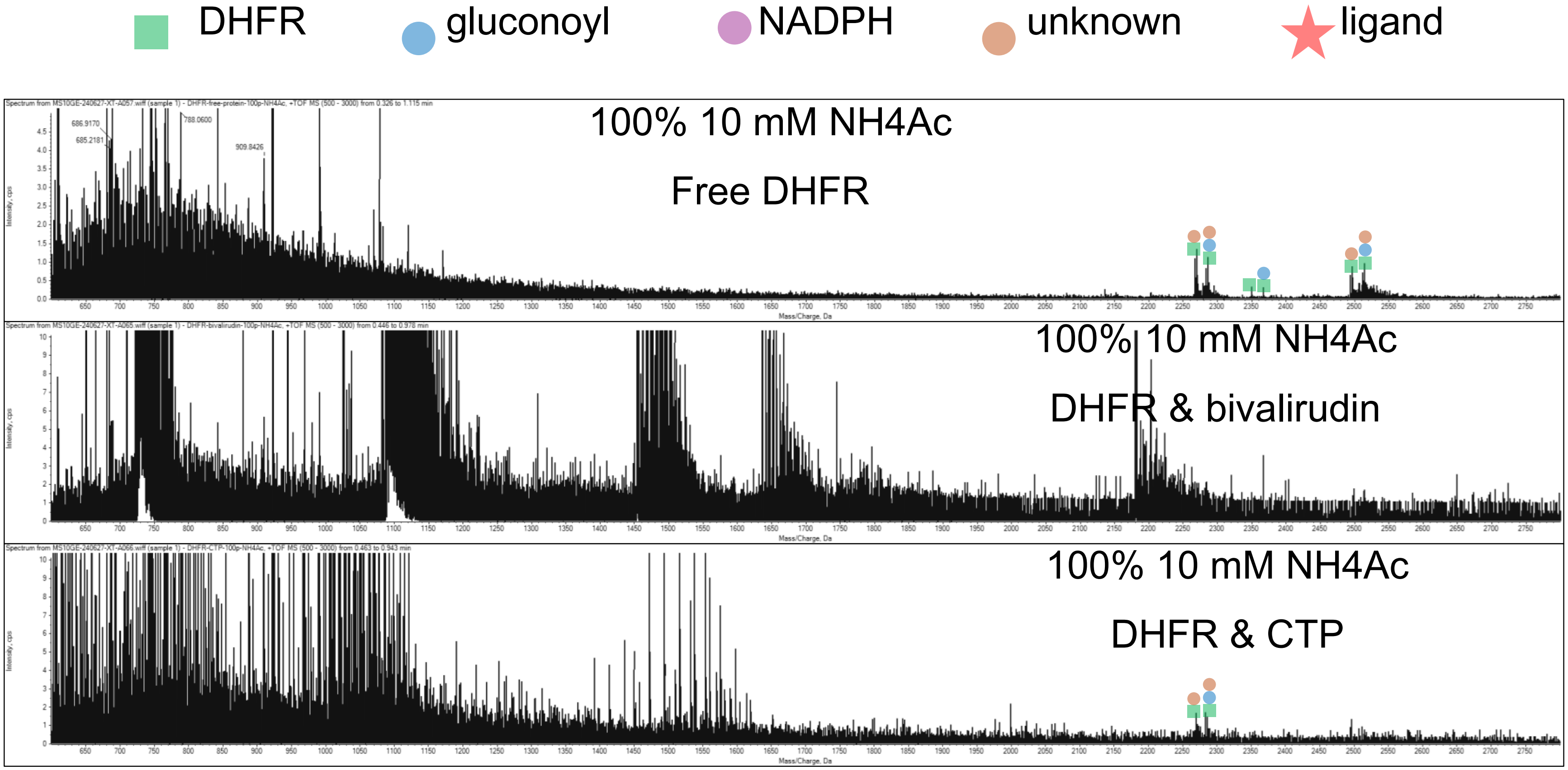
DHFR
gluconoyl
NADPH
unknown
ligand
100% 10 mM NH4Ac
Free DHFR
100% 10 mM NH4Ac
DHFR & bivalirudin
100% 10 mM NH4Ac
DHFR & CTP
Mass/Charge, Da
Intensity, cps

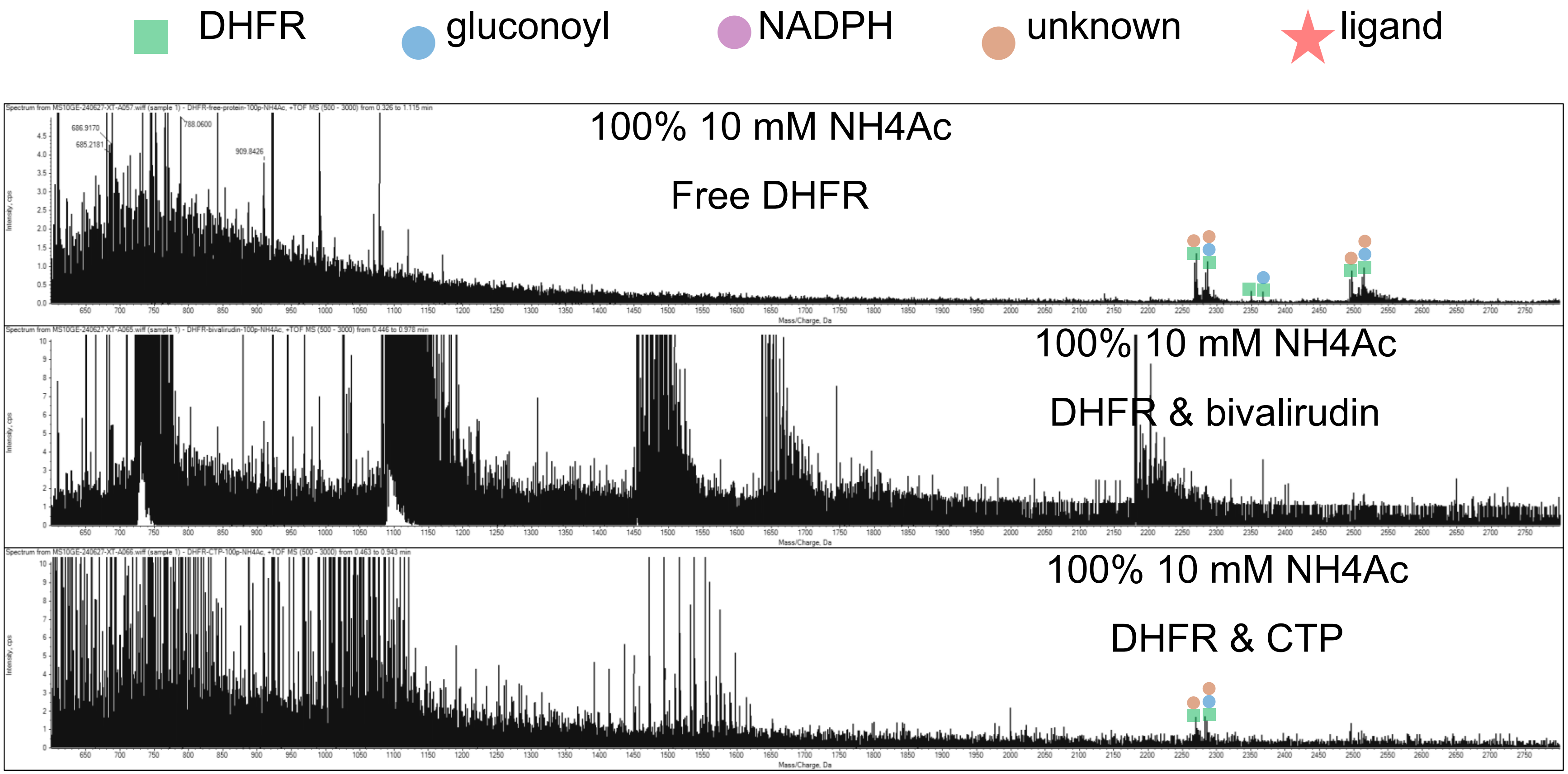

DHFR
gluconoyl
NADPH
unknown
ligand
100% 10 mM NH4Ac
Free DHFR
686.9170
685.2181
788.0600
909.8426
100% 10 mM NH4Ac
DHFR & bivalirudin
100% 10 mM NH4Ac
DHFR & CTP

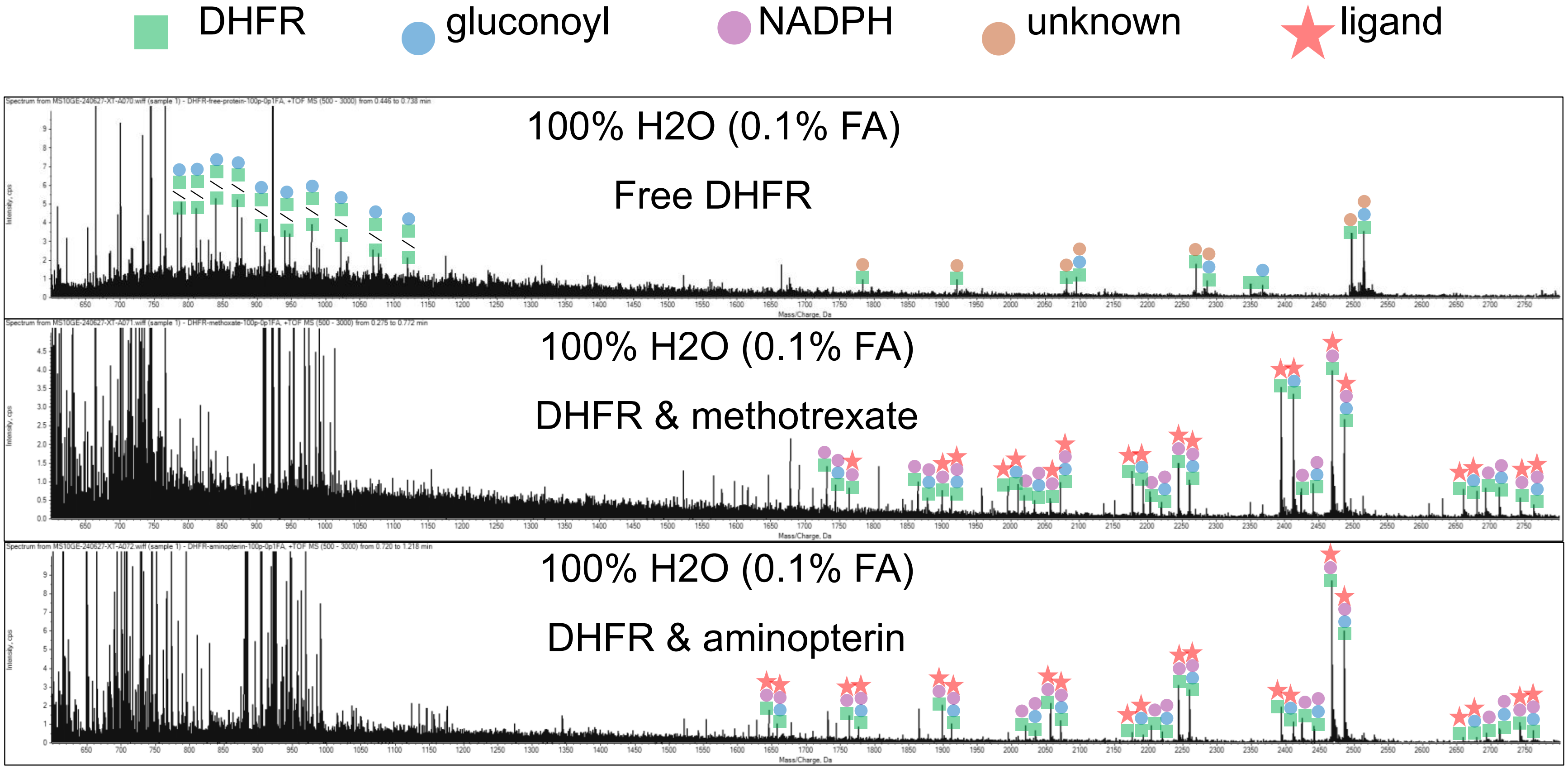
DHFR
gluconoyl
NADPH
unknown
ligand
100% H2O (0.1% FA)
Free DHFR
100% H2O (0.1% FA)
DHFR & methotrexate
100% H2O (0.1% FA)
DHFR & aminopterin
Mass/Charge, Da
Intensity, cps

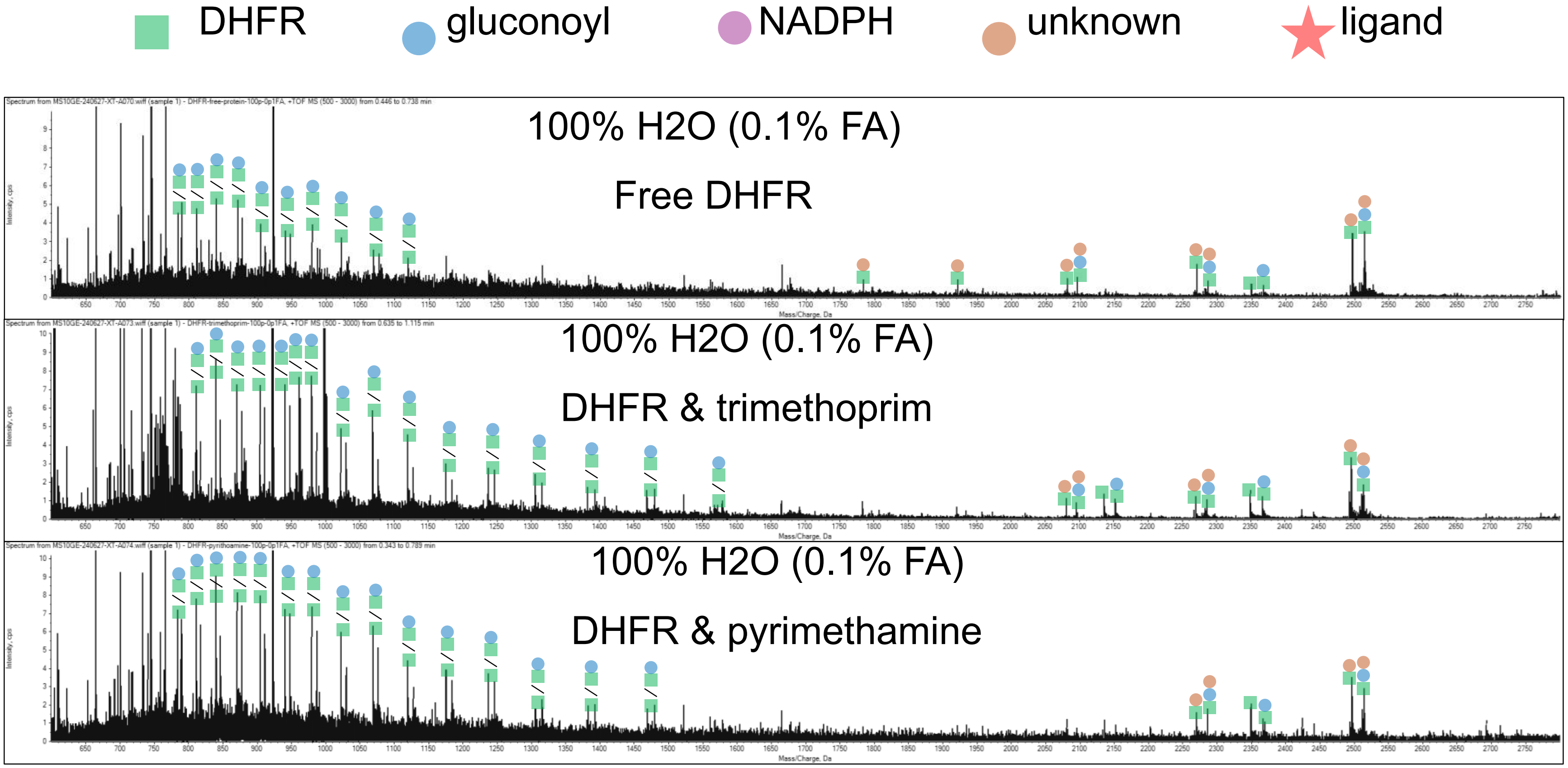
DHFR
gluconoyl
NADPH
unknown
ligand
100% H2O (0.1% FA)
Free DHFR
100% H2O (0.1% FA)
DHFR & trimethoprim
100% H2O (0.1% FA)
DHFR & pyrimethamine

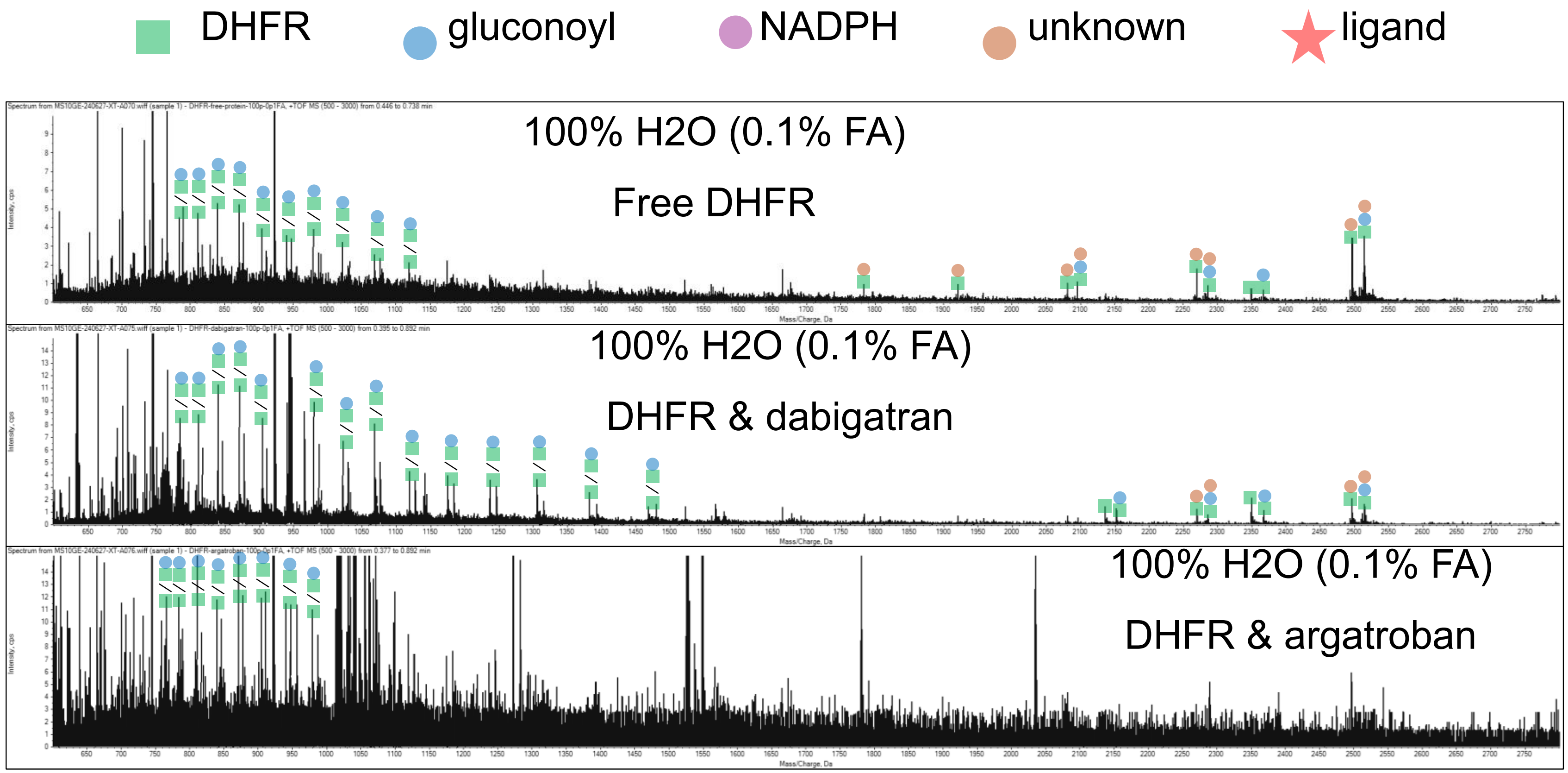
DHFR
gluconoyl
NADPH
unknown
ligand
100% H2O (0.1% FA)
Free DHFR
100% H2O (0.1% FA)
DHFR & dabigatran
100% H2O (0.1% FA)
DHFR & argatroban

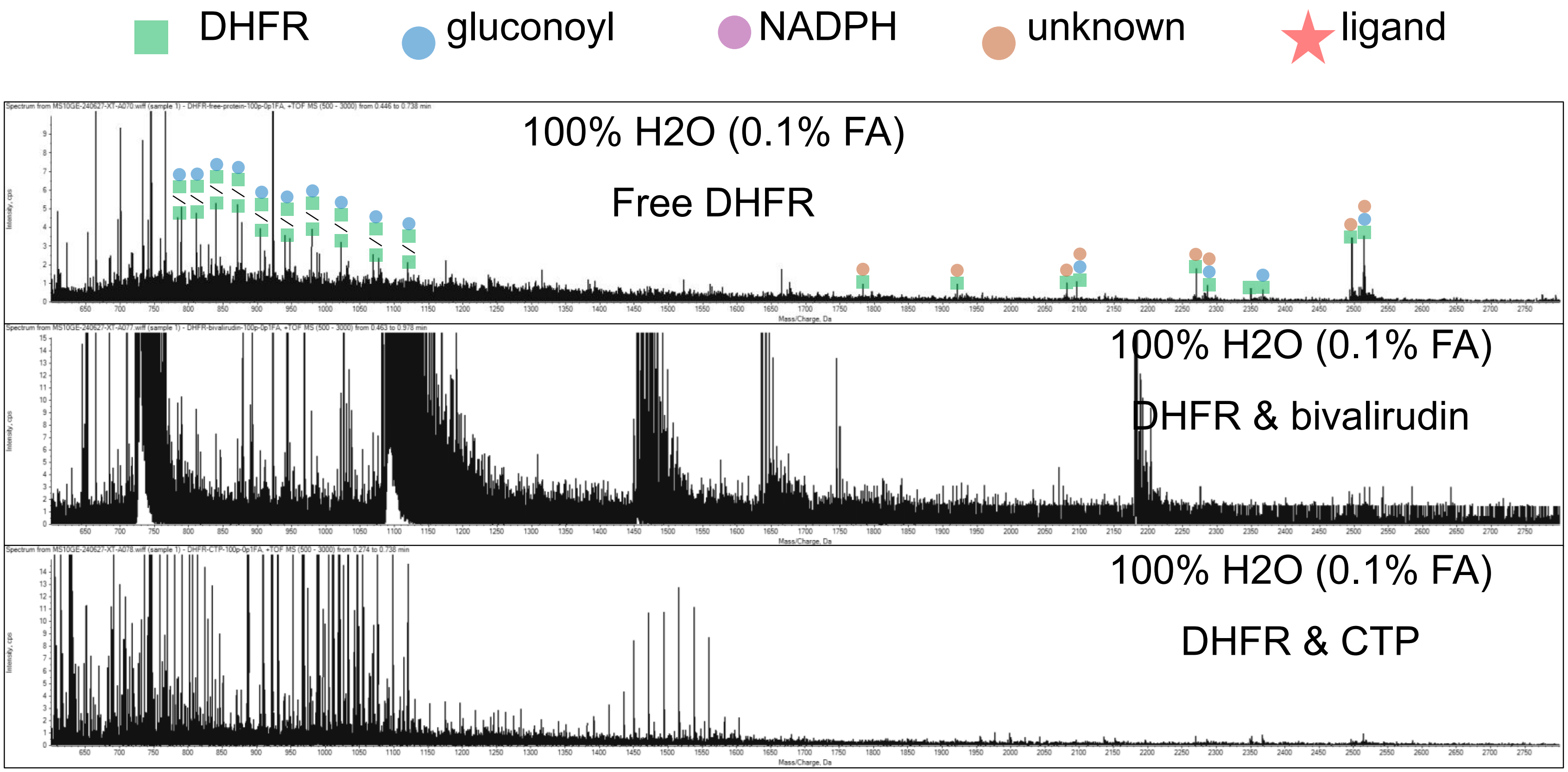
DHFR
gluconoyl
NADPH
unknown
ligand
100% H2O (0.1% FA)
Free DHFR
100% H2O (0.1% FA)
DHFR & bivalirudin
100% H2O (0.1% FA)
DHFR & CTP

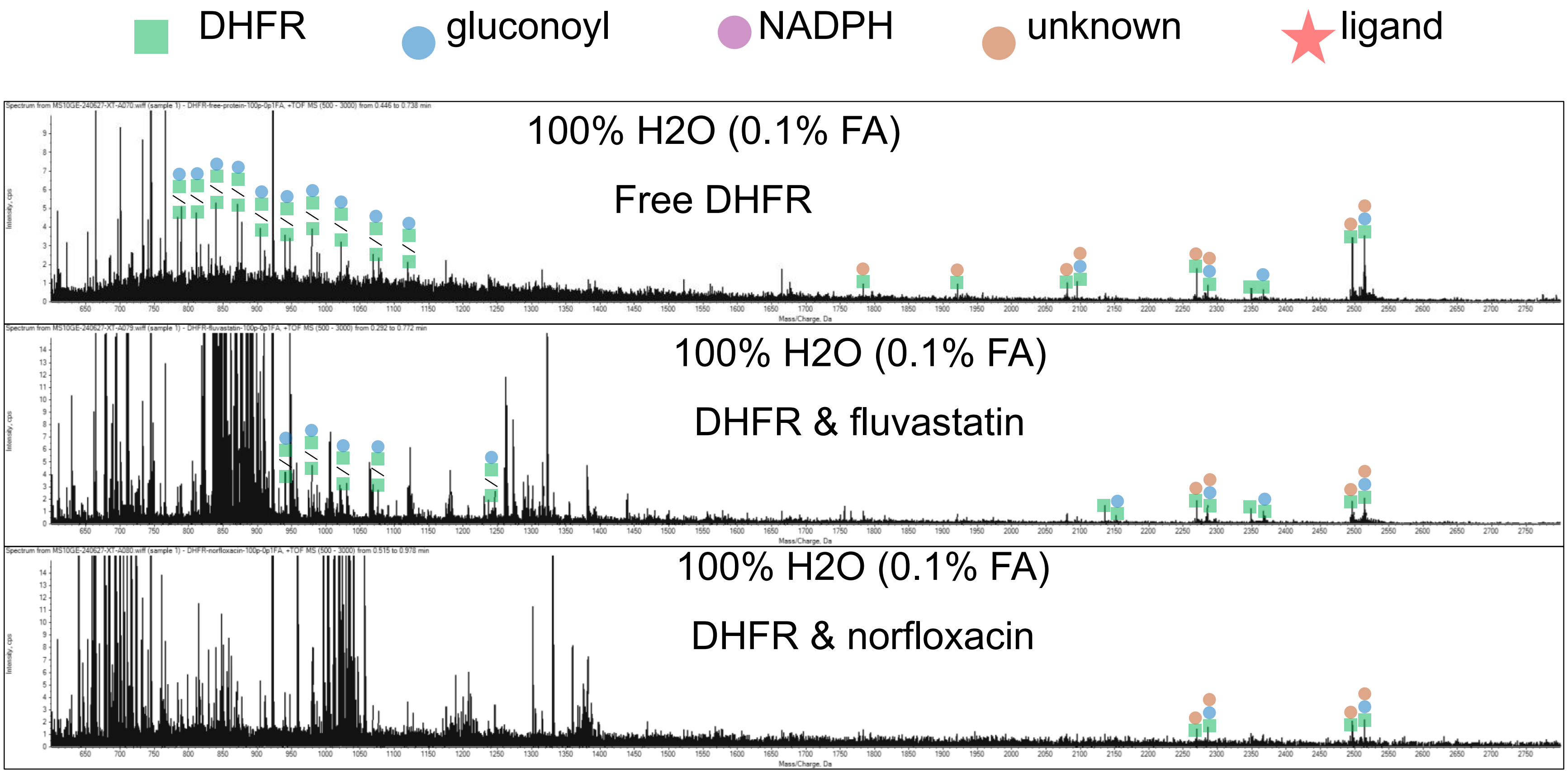

Figure S24 Original mass spectra of screening DHFR against 10 ligands under condition 1 (100% 10 mM NH_4Ac) and condition 2 (100% H_2O containing 0.1% FA).

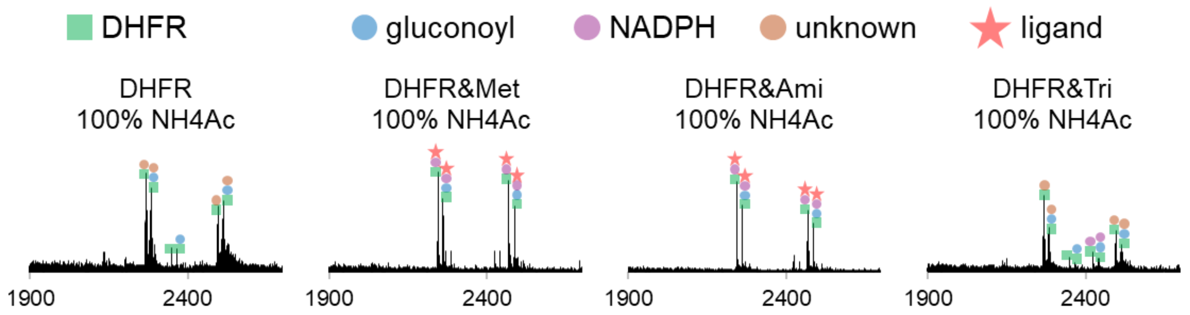

Figure S25. Raw mass spectra of free DHFR, DHFR & methotrexate, DHFR & aminopterin, and DHFR & trimethoprim of screenings under condition 1: 100% 10 mM NH4Ac.

DHFR sequence from abcam (supplier)

thrombin

MGSSHHHHHH SSG**LVPRGS**H MVGSLNCIVA VSQNMGIGKN
GDLPWPPLRN EFRYFQRMTT TSSVEGKQNL VIMGKKTWFS
IPEKNRPLKG RINLVLSREL KEPPQGAHFL SRSLDDALKL
TEQPELANKV DMVWIVGGSS VYKEAMNHPG HLKLFVTRIM
QDFESDTFFP EIDLEKYKLL PEYPGVLSDV QEEKGIKYKF
EVYEKND

Figure S26. Amino acid sequence of the recombinant human DHFR. The region highlighted with green is the digestion site of thrombin.

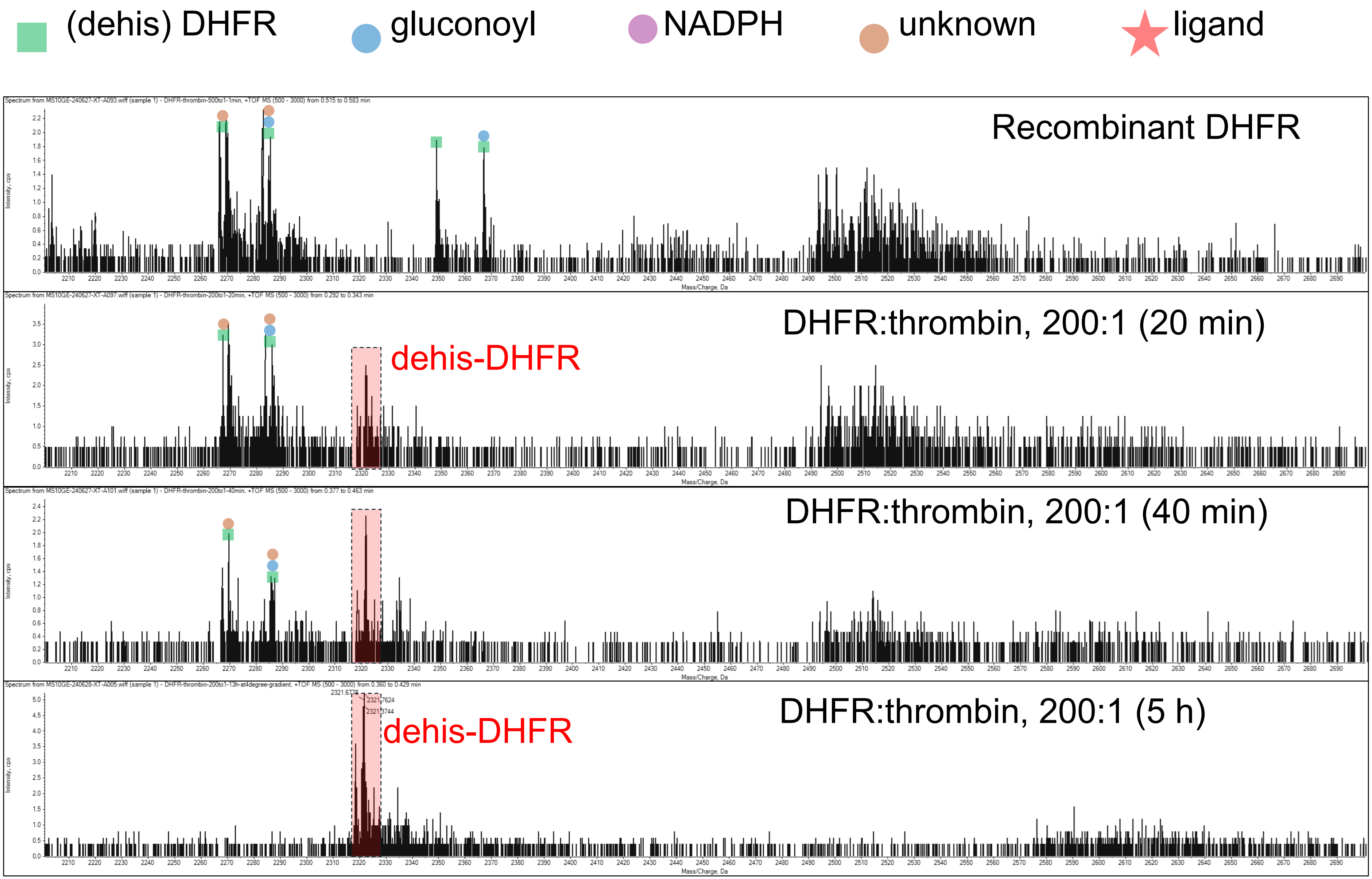

Figure S27. Tracking the loss of the his-tag from recombinant human DHFR with thrombin. His-tag was efficiently and specifically removed in 5 hours.

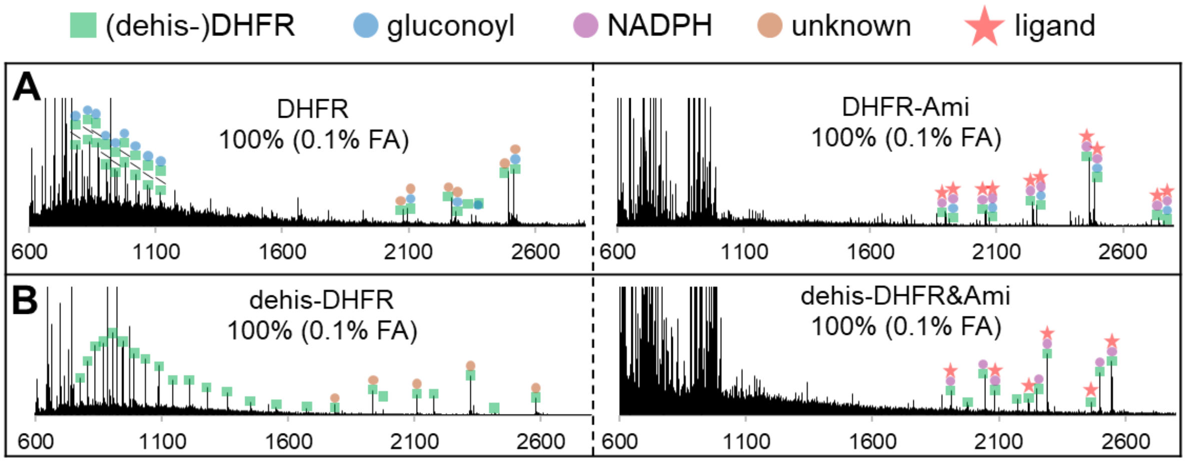

Figure S28. Raw mass spectra of free DHFR, DHFR & aminopterin, dehis-DHFR and dehis-DHFR & aminopterin under condition 6: 100% H_2O containing 0.1% FA. The green square, blue circle, purple circle, maroon circle and red star represent the protein (DHFR), the gluconoyl modification on the his-tag (178 Da), cofactor NADPH (744 Da), the unknown binder (1472 Da), and the ligand.
